# Immunological and pathological characteristics of brain parenchymal and leptomeningeal metastases from non-small cell lung cancer

**DOI:** 10.1038/s41421-025-00828-7

**Published:** 2025-08-29

**Authors:** Cheng Zhou, Shenbing Shan, Lei Wen, Da Liu, Changguo Shan, Xin Jin, Zhaoming Zhou, Hainan Li, Juan Li, Luyue Wang, Junguo Bu, Bin Li, Weishan Huang, Junhao Hu, Hongbo Guo, Wu Wei

**Affiliations:** 1 Department of Radiation Oncology, Nanfang Hospital, Southern Medical University, Guangzhou, Guangdong China; 2 Lingang Laboratory, Shanghai, China; 3 CAS Key Laboratory of Computational Biology, Shanghai Institute of Nutrition and Health, University of Chinese Academy of Sciences, Chinese Academy of Sciences, Shanghai, China; 4 Department of Radiation Oncology, Zhujiang Hospital, Southern Medical University, Guangzhou, Guangdong China; 5 Department of Neurosurgery, Guangdong Sanjiu Brain Hospital, Guangzhou, Guangdong China; 6 Department of Oncology, Guangdong Sanjiu Brain Hospital, Guangzhou, Guangdong China; 7 Department of Oncology, Nanjing First Hospital, Nanjing Medical University, Nanjing, Jiangsu China; 8 Department of Pathology, Guangdong Sanjiu Brain Hospital, Guangzhou, Guangdong China; 9 State Key Laboratory of Respiratory Disease, Key Laboratory of Biological Targeting Diagnosis, Therapy and Rehabilitation of Guangdong Higher Education Institutes, the Fifth Affiliated Hospital, Guangzhou Medical University, Guangzhou, Guangdong China; 10 Department of Pathobiological Sciences, School of Veterinary Medicine, Louisiana State University, Baton Rouge, LA USA; 11 Interdisciplinary Research Center on Biology and Chemistry, Shanghai Institute of Organic Chemistry, Chinese Academy of Sciences, Shanghai, China; 12 Department of Neurosurgery Center, The National Key Clinical Specialty, The Engineering Technology Research Center of Education Ministry of China on Diagnosis and Treatment of Cerebrovascular Disease, Guangdong Provincial Key Laboratory on Brain Function Repair and Regeneration, The Neurosurgery Institute of Guangdong Province, Zhujiang Hospital, Southern Medical University, Guangzhou, Guangdong China

**Keywords:** CNS cancer, Cancer microenvironment, Tumour immunology

## Abstract

Brain parenchymal metastases (BM) and leptomeningeal metastases (LM) represent distinct subtypes of central nervous system metastases (CNSm) from lung cancer, posing significant clinical challenges. The local immune landscape of LM remains elusive. Herein, we utilized single-cell RNA sequencing to build a cell atlas of LM, and systematically examine the immune profiling and cell heterogeneity between BM and LM. Our analysis reveals that BM has more CXCL9^+^ macrophages, CXCL13^+^CD4^+^ T cells and B cells than LM, exhibiting the presence of tertiary lymphoid (TLS) structures, which is associated with a favorable response to tyrosine kinase inhibitors (TKI). Conversely, a remarkably immunosuppressive tumor microenvironment (TME) is detected in LM, characterized by lymphocyte depletion and a concurrent enrichment of SPP1^+^ macrophages, compared to BM. Furthermore, we identified significant blood-brain barrier (BBB) cell discrepancies between BM and LM, and substantial phenotypic reprogramming of BBB cells in CNSm. This reprogramming encompassed alterations in transporter gene expression, extracellular matrix production and dysregulated cell-cell interactions, potentially contributing to the metastatic process. In summary, this study highlights the divergent cellular and molecular landscapes of BM vs LM, offering critical insights into potential therapeutic targets and informing the development of improved treatment strategies for non-small cell lung cancer patients with CSNm.

## Introduction

Central nervous system metastases (CNSm) have become increasingly prevalent due to extended survival of patients with non-small cell lung cancer (NSCLC), over half of them develop CNSm during the lifespan^1^. The incidence of brain parenchymal metastases (BM) at initial diagnosis of lung cancer was 28.6% and leptomeningeal metastases (LM) was 3%−5% in NSCLC patients^2,3^. In NSCLC, LM could occur either independently (36.6%), concurrent with (36.9%) or followed by (26.9%) brain parenchymal metastases^4^. Harboring drive gene mutations (i.e., *EGFR, ALK, ROS1*) are common high-risk factors for NSCLC patients to develop BM and LM^2,3^. Although both belong to central nervous system metastases, BM and LM show distinct differences, whether in anatomic location, clinical manifestation, therapeutic response, as well as long-term prognosis. In specific, LM is a fatal form of CNSm with a median survival of 3−6 months, compared to 5−15 months in BM^5,6^. However, there remains lacking of effective treatment for LM. The resistant mechanisms to multiple treatment strategies, i.e., immunotherapy with PD(L)-1 blockades, targeted therapy and radio- or chemotherapy are poorly understood. Hence it is critical to explore microenvironmental perturbations in context of metastasis particularly for LM.

Previous studies on BM revealed a prevalent presence of exhausted CD8^+^ T cells, which work in coordination with a substantial infiltration of tumor associated macrophages (TAM) to reinforce immune-suppressive environments^7,8^. The extensive infiltration of TAM highlights the potential for targeting TAM through CSF1R inhibition as a strategy to reduce BM progression^9^. Furthermore, it is noteworthy that cancer cells in BM adapted to various features associated with proliferative, inflammatory and even neuronal-like characteristics, enabling their proliferation within brain parenchyma^10,11^. On the other hand, studies on LM mainly relied on mouse models as well as analysis of cerebral spinal fluid (CSF)^12^. Single-cell RNA sequencing (scRNA-seq) based on CSF of patients suggested that LM was featured with immune-suppressed T-cells^13^ and lipid-associated macrophages (RNASE1^+^ macrophages)^14^. According to scRNA-seq and cell-free DNA-sequencing (cfDNA-seq) on CSF samples, increased CD8^+^ T cells infiltration and INFγ level were increased after immune check blocking (ICB) therapy^15^, but minimal long-term responders were observed in LM^13^.

Relying on mouse models and human CSF samples, recent studies have progressed in understanding the mechanisms of leptomeningeal metastases. Boire et al. reported that lung and breast cancer cell derived complement component 3 (C3) activated the C3a receptor in the choroid plexus epithelium to disrupt the blood-CSF barrier and interruption of C3a receptor signaling blocks LM in mice^16^. Macrophages in CSF can promote cancer cells producing lipocalin-2 (LCN2) for tumor growth^17^. Breast cancer cells can co-opt intrinsic neuronal-stroma cross-talk to thrive in the LM, while tumor cells closely interacted with NCAM^+^ meningeal macrophages for providing a combined physical and molecular shield against cytotoxic stressors^18^. A recent study revealed that dura-derived LM-associated macrophages (dLAMs) contribute to LM progression, and inhibiting SPP1-MMP14 axis can limit dLAMs migration into the CSF and thus prevent cancer cell growth and survival within mouse meningeal space^19^.

In fact, there are two phenotypes of LM cancer cells: floating cells within the cerebrospinal fluid (CSF) and cells that are adhesive to the leptomeninges^20^. Little is known about the immune microenvironment of adhesive LM metastasis in situ from human specimen. Herein, we have successfully collected leptomeningeal tissues from persistent intracranial hypertension patients with lung cancer or melanoma that greatly facilitate the pathological diagnosis of the disease^21,22^. The present study depicted the landscape of TME in LM, BM and their paired primary cancer tissues at single-cell level, and systematically compared the immune profiles as well as stromal cells that involved in shaping clinical discrepancies between BM and LM.

## Results

### CNSm landscape of NSCLC displays significant TME

**Fig. 1:**
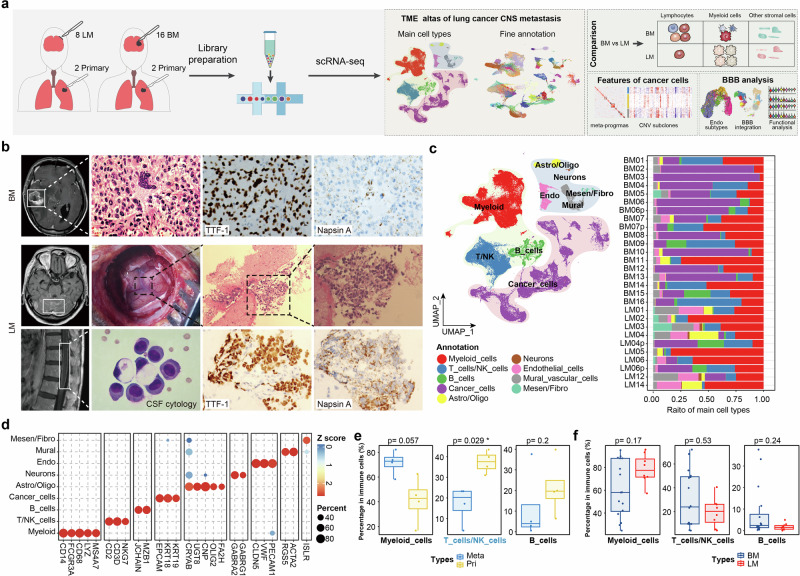
Characterization of the BM and LM TME by single-cell RNA sequencing. **a** Schematic overview of the study design, including sample types, data compositions, and analytical workflows. **b** Representative radiological and pathological features of brain metastases. Top panel: T1-weighted contrast-enhanced magnetic resonance imaging (MRI) and hematoxylin and eosin stain (H&E) staining of formalin-fixed paraffin-embedded (FFPE) tissues for brain parenchyma metastases (BM), as well as TTF-1 and Napsin A immunostainings (40×) for primary lung adenocarcinoma from patient BM02 (BM02p). Lower two panels, characteristics of leptomeningeal metastases (LM) (sample from LM04). Middle panel, from left to right: diffuse linear enhancement in T1-weighted contrast-enhanced MRI of the brain; the surgical view; representative histopathological images of the intraoperative frozen section (H&E; 20× and 40×). Bottom panel, from left to right: diffuse linear enhancement in T1-weighted contrast-enhanced MRI of the spine; massive tumor cells were found in cerebrospinal fluid (CSF) in the representative patient diagnosed with LM (H&E staining); TTF-1 and Napsin A IHC staining (40×) of primary lung cancer from patient LM04 (LM04p). **c** UMAP visualization of main cell types, colored by cell types (left panel) and main cell type abundance in each sample (right panel). Mesen/Fibro, mesenchymal cells/fibroblasts; Mural, mural cells; Endo, endothelial cells; Astro/Oligo, astrocytes/oligodendrocytes; T/NK cells, T cells or nature killer cells; Myeloid, myeloid cells. Samples with names ending with “p” are from primary cancers. **d** The expression of marker genes for main cell types. Heatmap color indicates the level of gene expression scaled by columns, and dot size indicates the percentage of cells expressing the genes in different cell types. **e**, **f** Comparisons of percentages of immune cell types between primary and metastatic samples (**e**), and between BM and LM samples (**f**). *p*-value was calculated by the Wilcoxon test. Meta metastasis cancer, Pri primary cancer.

To elucidate the CNSm transcriptional landscape at single-cell resolution, BM (*n* = 16) and LM (*n* = 8) fresh tissue samples from 24 NSCLC patients were collected. Additionally, paired primary lung cancer tissue were also collected in 2 patients with BM and 2 with LM. Notably, the primary tissue in the study refers to the primary tumor, not a recurrence. All LM samples were derived from patients’ leptomeningeal tissues. Therefore, this study focused on adhesive LM tumor cells (Fig. 1a; Supplementary Table S1, and Video S1). The diagnoses of CNSm were confirmed via brain MRI, resected brain tumor pathology, or cerebrospinal fluid (CSF) cytology. Immunohistochemistry (IHC) staining of TTF-1 and Napsin A, two markers of lung adenocarcinoma, validated that both BM and LM originated from lung cancer (Fig. 1b). After preprocessing and batch correction, the scRNA-seq generated more than 190,000 high-quality cells for further analysis (Materials and methods; Supplementary Fig. S1a, b). The scRNA-seq data were analyzed using Seurat standard pipeline and visualized by uniform manifold approximation and projection (UMAP)^23^, followed by main cell type annotation (Fig. 1c) based on classical cell markers (Fig. 1d; Supplementary Fig. S1c and Table S2).

Most cell types organized into continuous clusters, but tumor cells from different patients formed multiple discrete clusters, and the differences were further shown by lower LISI scores in cancer cells, reflecting the heterogeneity of tumor cells, revealed significant inter- and intra-patient heterogeneity in cancer cell transcriptomes (Supplementary Fig. S1d, e), consistent with previous studies^11^. Although the percentage of immune cells is similar among different groups (Supplementary Fig. S1f, g), considerably reduced infiltration of T/NK and B cells as well as increased myeloid cells were observed in CNSm, particularly LM compared to primary tissues (Fig. 1e, f; Supplementary Fig. S1h, i, and Table S3), indicating an immunosuppressive environment in CNSm. The main cell type differences between BM and LM were further validated by Cacoa (Supplementary Fig. S1j), a case-control analysis tool for comparative designs^24^. To dissect the microenvironment of CNSm more precisely, we further sub-divide into main cell types into 69 clusters, encompassing immune, cancer, and other stromal cells at a high resolution (Supplementary Fig. S1k). Overall, the scRNA-seq analysis revealed significant inter- and intra-patient heterogeneity in the transcriptional landscape of lung cancer CNS metastases, highlighting a more pronounced immunosuppressive environment in CNS metastases than in primary lung cancers.

### CD8^+^ T cells are exhausted in CNSm of NSCLC

**Fig. 2:**
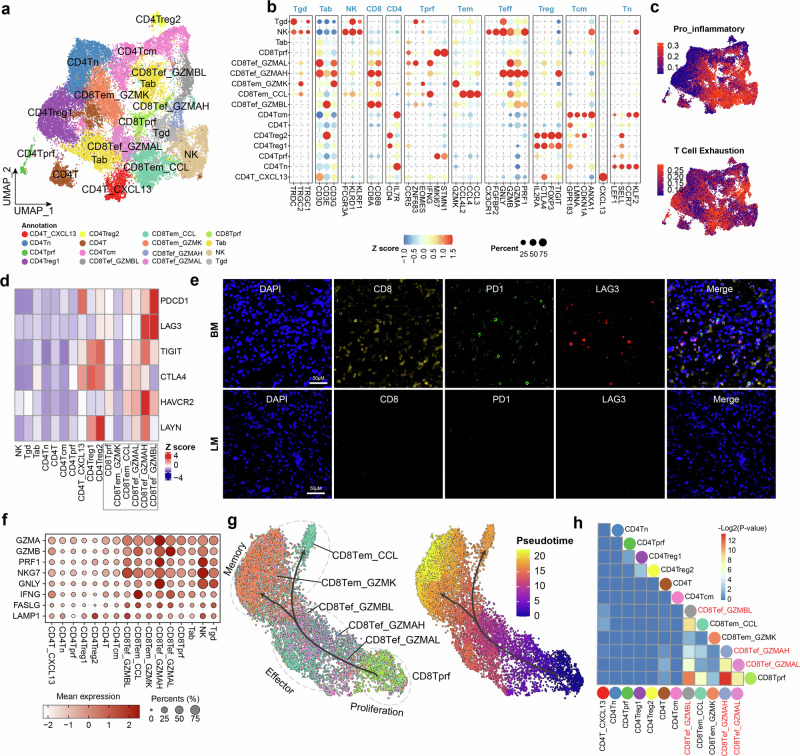
Characterization of T/NK cells in brain metastases. **a** UMAP visualization of T/NK cells, colored by cell clusters. **b** The expression of marker genes for different subsets of T/NK cells. The color indicates relative gene expression, and dot size indicates the percentage of cells expressing the indicated genes. **c** UMAP visualization of T/NK cells, colored by signature scores calculated by AUCcell. **d** Expression of gene signatures of T cell exhaustion by different subsets of T/NK cells. The mean gene expressions were normalized to the overall gene expression. **e** Multiple channel immunohistochemistry (mIHC) staining of indicated genes in BM and LM. **f** The expression of effect, cytotoxic/memory-related genes in T/NK cells. **g** The trajectory of CD8^+^ T cell transition inferred by Monocle3. Left panel: inferred trajectory layout of CD8^+^ T cells. Cells were colored as in (**a**). Three main cell states (proliferation, effector, and memory) were marked by a black dashed oval box. Right panel: pseudotime of the inferred trajectory of CD8^+^ T cell development. **h** The heatmap showing the connectivity between T cell clusters based on shared clones. The *p* values were calculated by hypergeometric distribution. Effector cells were colored in red.

In CNSm of NSCLC, main T/NK cells types were annotated into 16 cell states according signature genes of T/NK cells, including two CD8^+^ effector memory clusters (CD8Tem_CCL and CD8Tem_GZMK) and three CD8^+^ effector clusters (CD8Tef_GZMBL, CD8Tef_GZMAL and CD8Tef_GZMAH), exhibiting extensive heterogeneity of tumor-infiltrating T cells (Fig. 2a, b; Supplementary Fig. S2a). Proinflammatory features were high in CD8^+^ T cells, and T cell exhaustion scores also were detected among these cells (Fig. 2c). The majority of CD8^+^ T cells as well as CD4T_CXCL13 cells express exhaustion markers (Fig. 2d). Specifically, CD8Tef_GZMBL and CD4T_CXCL13 were the main cell clusters expressing high levels of *PD1*, potentially influencing patient responses to anti-PD1 therapy. Of note, *PD1* expression was lower than *LAG3* and *TIGIT* in CD8^+^ T cells (Supplementary Fig. S2b), suggesting that *LAG3* and *TIGIT* might be potential targets for alternative immunotherapies in lung cancer CNSm. To validate the exhaustion states of CD8^+^ T cells in CNSm, multiple channel immunohistochemistry (mIHC) staining was performed to detect CD8, PD1 and LAG3. Consistent with results of scRNA-seq analysis, PD1 or LAG3 positive CD8^+^ T cells were widespread in BM, while LM had minimal CD8^+^ T cells (Fig. 2e). Moreover, despite the expression of effector molecules such as *GZMA*, *NKG7* and *GZMB* (Fig. 2f), these CD8^+^ T cells lacked expression of proinflammatory cytokines *IL2* and *TNF* (Supplementary Fig. S2c). Our results suggest that CD8^+^ T cell exhaustion is prevalent in CNSm from NSCLC and the different exhaustion levels exist in the metastatic environment.

We then investigated the development path of these CD8^+^ T cells, especially for exhausted CD8^+^ T cells. Pseudotime analysis revealed that CD8Tef_GZMAL, CD8Tef_GZMAH, and CD8Tef_GZMBL were positioned progressively farther from the starting point, in line with their increasing levels of exhaustion (Fig. 2g; Supplementary Fig. S2d). The expression of naïve/memory T markers including *TCF7* and *CCR5*^25^, was completely absent in effector CD8^+^ T cells (Supplementary Fig. S2e), implicating a severer exhaustion status in CNSm. Divergent developmental paths and enriched gene ontology (GO) terms of differentially expressed genes were identified between CD8Tem_CCL and CD8Tem_GZMK, which suggested that CD8Tem_CCL may represent an alternative cell state with different biological functions from CD8Tem_GZMK (Fig. 2g; Supplementary Fig. S2f, g).

Seven samples had also been performed with TCR sequencing simultaneously. Our TCR analysis show that most TCR sequences were shared by only a single cell (Supplementary Fig. S3a). The clonal expansion capacity varied among different T cell clusters, with CD8^+^ T cells generally exhibiting stronger expansion than CD4^+^ T cells (Supplementary Fig. S3b, e). Notably, we identified two hyperexpanded clones within the CD8^+^ T cell population (Supplementary Fig. S3f, g). These results are consistent with previous reports^26^.

To further explore the lineage relationships of T cell clusters, we analyzed their shared T cell-receptor (TCR) sequences, finding that CD8Tprf (proliferation CD8 T cell) shares significant TCR sequences with all CD8^+^ T cells (*p* < 0.05) and that three exhausted CD8^+^ T cell clusters were also highly related (Fig. 2h; Supplementary Fig. S3h, i). By integrating scRNA-seq and scTCR-seq results, we identified a potential developmental path where CD8Tprf cells could differentiate into various CD8^+^ T subtypes, particularly effector CD8^+^ T cells, as indicated previously^27^. Collectively, our findings suggest the rarity of naïve CD8^+^ T cells and extensive exhaustion of CD8^+^ effector T cells, indicating limited recruitment of CD8^+^ T cells in CNSm, with infiltrated cells experiencing prolonged antigenic stimulation. Given that NK cells represent the terminal effector cells of the innate immune system at metastatic sites^28,29^, we performed further sub-clustering of these cells (Supplementary Fig. S3j, k), and found differential expression of effector molecules and dynamic trajectories among NK clusters (Supplementary Fig. S3l−o). These results indicate that NK cells also serve as key contributors of cytotoxic mediators to perform anti-tumor roles.

### Rare lymphocytic infiltration in LM

**Fig. 3:**
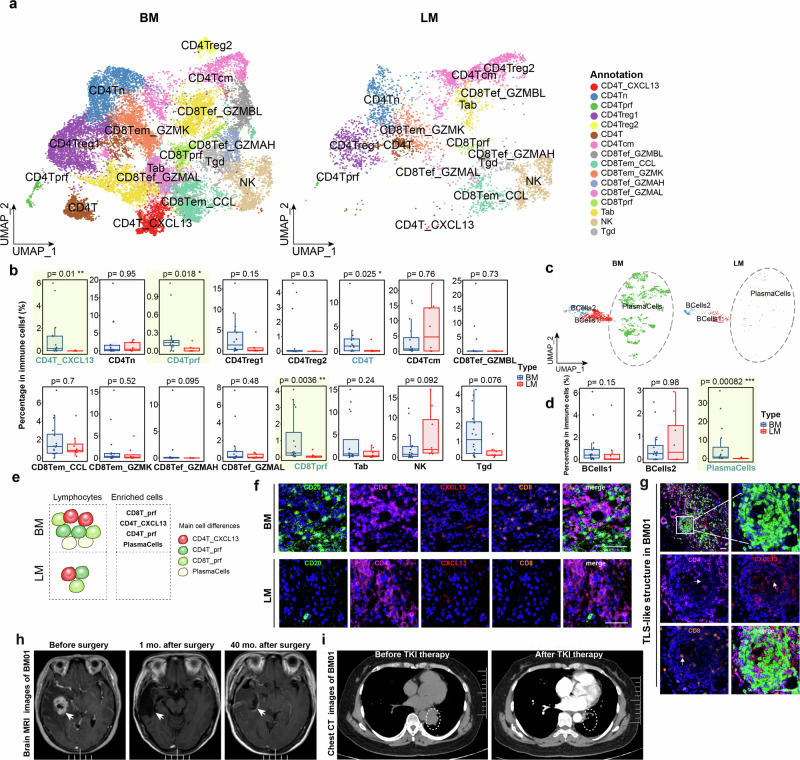
Less lymphocyte infiltration in the TME of LM than BM. **a** Comparison of T/NK cell clusters between BM and LM. **b** Comparison of the percentages of T/NK cell clusters between BM and LM. Cell percentages were calculated by dividing the number of each cell state by the total number of immune cells. Main cell differences between BM and LM were highlighted with yellow transparent background. **c** Comparison of UMAP visualization of B cell clusters between BM and LM, colored by cell clusters. Plasma cells were marked by grey dashed circles. **d** Cell percentage of B cell clusters between BM and LM. **e** A sketch of the differences in lymphocyte composition between BM and LM. **f** mIHC staining of selected marker genes in BM and LM. Scale bar: 50 μm. **g** Selected marker genes show a TLS-like structure in BM01. Scale bar: 50 μm. **h** Brain MRI images before, 1 month, and 40 months after surgery with tumor resection of BM01. **i** Representative Computed Tomography (CT) images before and after treatment with EGFR-TKI (Osimertinib) of BM01.

While comparing the T/NK cell compositions between BM and LM, we found that all subpopulations of CD8^+^ T cells infiltration were considerably reduced in LM compared to BM (Fig. 3a; Supplementary Table S4). For example, the mean proportions in all immune cells of CD8Tprf in LM were significantly lower than in BM, and the reduction of CD8Tprf may cause impaired CD8^+^ lineage development capacity in LM. Likewise, there was a significantly higher percentage of CD4T_CXCL13 cells, a good indicator for effective responses to PD-L1 blockade^30^, and CD4Tprf infiltration in BM than in LM (Fig. 3b). A significantly lower abundance of plasma cells was observed in LM than in BM (Fig. 3c, d). Furthermore, metastatic lesions exhibited considerably less T/NK and B cells infiltration than their primary lung cancer tissues (Supplementary Fig. S4a−g). To validate the reduced lymphocyte infiltration in LM (Fig. 3e), mIHC staining found a significantly increased proportion of CD8^+^ T and B cells in BM than in LM (Fig. 3f; Supplementary Fig. S4h−i). Interestingly, we observed that CD4_CXCL13 formed tertiary lymphoid structures (TLS) with B cells and CD8^+^ T cells (Fig. 3g). It suggests that CD4_CXCL13 might interact with CD8^+^ T cells and B cells in the TME to modulate local tumor immunity in BM. Notably, the patients with TLS-like structures experienced no tumor relapse for 40 months post-surgical resection (Fig. 3h), associating with a significant reduction in primary tumor volume following Osimertinib treatment (Fig. 3i). These findings indicate that LM lacked lymphocytic infiltration, especially CD8^+^ T cells and plasma cells, suggesting an intensively immunocompromised TME in LM than BM, attributing to a poorer response to immunotherapy.

### SPP1 positive macrophages are dominant in LM

**Fig. 4:**
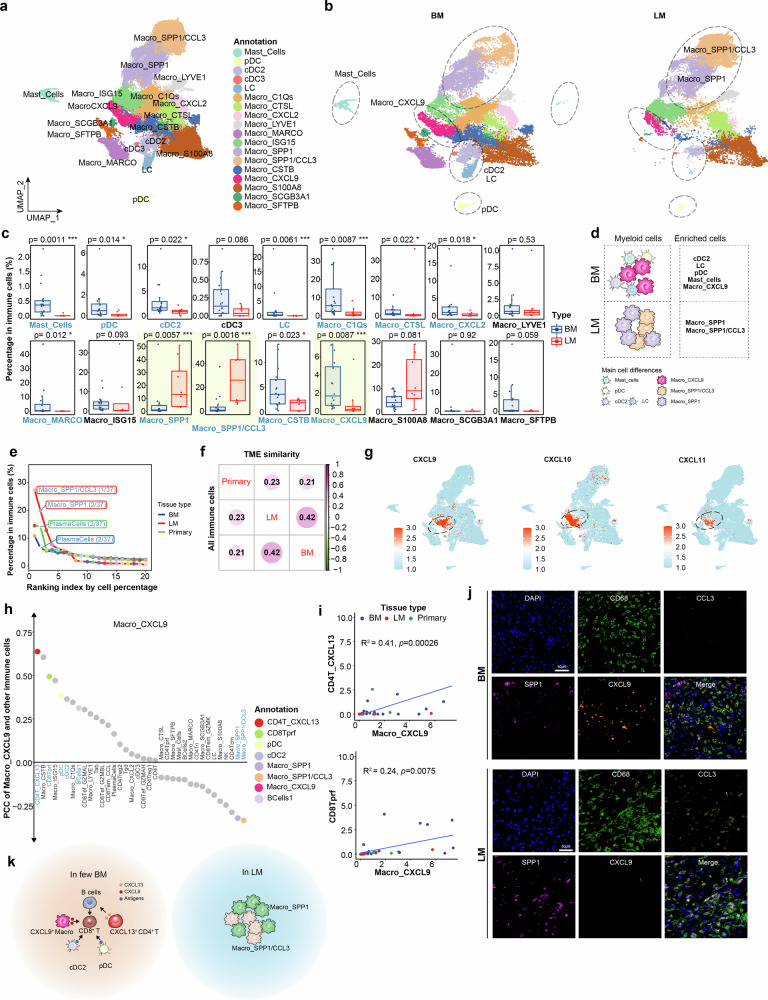
SPP1 positive macrophages are dominant myeloid cells in LM. **a**, **b** UMAP visualization (**a**) and comparison (**b**) of myeloid cells, colored by myeloid cell clusters. Main myeloid cell differences were marked by black dashed circles. **c** Cell percentage differences of myeloid cell clusters between BM and LM. Cell percentages were calculated by dividing the number of each cell state by the total number of immune cells. Main cell differences highlighted with yellow transparent background. **d** Schematic diagram of the difference in myeloid cell composition between BM and LM. **e** Immune cell compositions of BM, LM, and primary lung cancer. Cell clusters were ranked according mean cell percentage in each tissue type. Points were colored by cell clusters, while lines were colored by tissue type. Top cell clusters in each tissue type were marked in labels and ranking index. **f** TME similarity among BM, LM, and primary lung cancer in all immune cells. The size of the circles and the intensity of the color indicate the magnitude of the similarity, calculated by the Kendall coefficient. **g** Expression of *CXCL9/10*/*11* in all myeloid cells. **h** The correlation of the relative abundance between Macro_CXCL9 and all other immune cell clusters. Representative cell clusters are highlighted. **i** The correlation of the percentage of Macro_CXCL9 and CD4T_CXCL13 (upper panel) and CD8Tprf (lower panel). Each point represents one sample, and points were colored by tissue type. **j** mIHC staining of indicated genes in BM and LM. **k** Schematic overview of the difference in immune cell profiles in the TMEs between BM and LM.

Myeloid cells could also participate in shaping the TME of LM and BM. In line with findings in other tumors^31^, highly heterogeneous myeloid cells were also observed in CNSm (Fig. 4a; Supplementary Fig. S5a). Accordant to the infiltration patterns of lymphocytes, macrophage clusters (including Macro_CXCL9), mast cells and most DCs were significantly abundant in BM compared to LM. Conversely, Macro_SPP1 and Macro_SPP1/CCL3 cells were substantially enriched in LM compared to BM (mean proportions 19.85% vs. 5.31% and 27.27% vs. 5.80% in all immune cells, respectively) (Fig. 4b, c). Furthermore, antigen presentation genes such as *HLA-DQA1*, were highly expressed in BM, while immune inhibitory genes such as *IL10* and *VEGFA* were upregulated in LM (Supplementary Fig. S5b), suggesting extensive myeloid dysfunction with respect to antigen presentation and immune modulation. The distinct compositions of myeloid cells between LM and BM suggested a defective antigen presentation capacity for T cells and enriched infiltrated SPP1^+^/CCL3^+^ macrophages in LM (Fig. 4d). We found that these SPP1 positive myeloid cells (Macro_SPP1/CCL3 and Macro_SPP1) resembled the pro-tumor macrophages in a CXCL9-SPP1 macrophage polarity system, a new human cancer dataset based classifying system to replace M1-M2^32^. In addition, SPP1^+^ macrophages have been shown to be anti-inflammatory tumor-associated macrophages (TAMs)^33,34^.

We then ranked immune subtypes according their abundances to systematically compared the TME differences among BM, LM and primary lung cancer in whole immune cell level. We found that Macro _SPP1/CCL3 and Macro_SPP1 cells dominated the LM samples (nearly 50% of the LM-infiltrating immune cells). In contrast, BM was abundant for plasma cells, similar to primary cancer samples (Fig. 4e). Interestingly, The Macro_SPP1/CCL3 cells were able to interact with other macrophage subsets via CCR1 (Supplementary Fig. S5c), involved in recruitment and retention of TAMs^35^, consistent with the higher myeloid cell infiltration in LM. Moreover, BM and LM had higher overall immune cell similarity than primary tissues (Fig. 4f), whereas lymphocyte similarity was higher between BM and primary cancers (Supplementary Fig. S5d). These results highlight significant differences in TME, particularly in lymphocytes and myeloid cell subtypes, between BM and LM. These findings suggest that stratified treatments targeting distinct TMEs for LM and BM may be considered in the future.

### Multiple cell subtypes involved in contributing to pro-inflammatory environment in BM

The pro-inflammatory environment in BM is characterized by the presence of CXCL9^+^ myeloid cells, which are reported to have anti-tumor properties^32^. In CNSm samples, Macro_CXCL9 cells expressed *CXCL9*, *CXCL10*, *CXCL11* (Fig. 4g), which are capable of recruiting CD8^+^ T cells. The abundance of Macro_CXCL9 cells correlated positively with CD8Tprf cells, CD4T_CXCL13 cells and multiple DCs subsets (Fig. 4h, i). Furthermore, cell−cell communication analysis^36^ revealed that Macro_CXCL9 cells could communicate with multiple CD8^+^ T subtypes via *CXCR3/6* receptors (Supplementary Fig. S5e). Meanwhile, CD4T_CXCL13 cells correlated well with multiple B cells and CD8^+^ T cells (Supplementary Fig. S5f, g). More interestingly, both Macro_CXCL9 and CD4T_CXCL13 correlated inversely with *SPP1* positive cells (Fig. 4h; Supplementary Fig. S5g). The mIHC results showed that BM was enriched with CXCL9^+^ macrophages, while LM had almost no CXCL9^+^ macrophages but contained plenty of CCL3^+^ and SPP1^+^ macrophages (Fig. 4j). We also utilized gene signatures from the published study to score cells for their association with T-cell expansion^37^. Our analysis revealed that Macro_CXCL9 macrophages exhibited the highest scores for genes positively correlated with T cell expansion, while in contrast, the Macro_SPP1/CCL3 and Macro_SPP1 macrophages scored highest for genes negatively associated with T-cell expansion (Supplementary Fig. S5h).

Moreover, we performed spatial transcriptomics on an BM sample. The spatial analysis has revealed that the niche including CXCL9^+^ macrophages also showed expression of T-cell markers, consistent with the positive correlation between CXCL9^+^ macrophages and CD8^+^ T cells in scRNA-seq results. More importantly, we discovered a distinct spatial organization: CXCL9^+^ macrophages were predominantly localized to the periphery of the tumor, whereas SPP1^+^ macrophages were situated closer to the cancer cells (Supplementary Fig. S5i−l). All of these results reveal that Macro_CXCL9 cells together with CD4T_CXCL13, B cells and DCs contribute to building a pro-inflammatory microenvironment in BM. In contrast, LM is dominated by immunosuppressive SPP1^+^ macrophages, explaining why LM patients are associated with a poor treatment response compared to BM (Fig. 4k).

### Characteristics of cancer cells in the CNSm TME

**Fig. 5:**
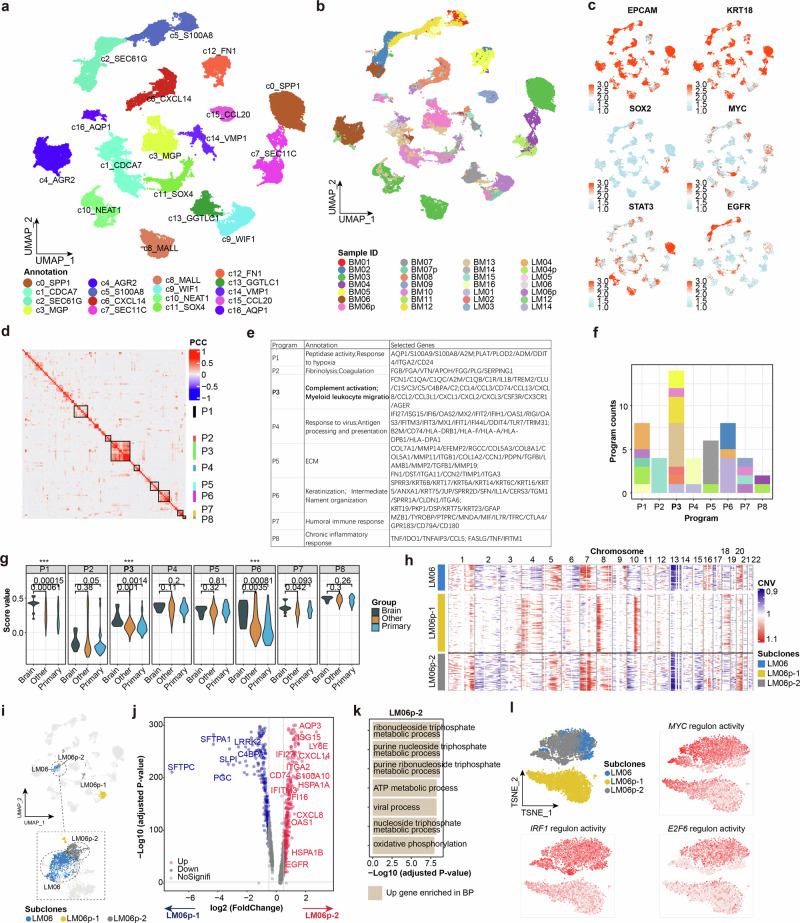
Characteristics of lung cancer cells in CNSm. **a**, **b** UMAP visualization of all cancer cells, colored by clusters (**a**) and sample IDs (**b**). **c** Expression of representative genes in cancer cells. **d** Meta-programs of cancer cells. **e** Annotation and represented genes for meta-programs. **f** The compositions of meta-programs. Colored by sample ID as in **b**. **g** The meta-program scores of the MET500 dataset among CNSm, other metastatic tissues, and primary tissues. **h** CNV patterns of LM06 and its paired primary sample LM06p. **i** Highlighting subclones from patient LM06 in UMAP. **j** Differential gene expression analysis between LM06p-2 and LM06p-1. Criteria: log2 fold change ≥ 0.5, Benjamini-Hochberg adjusted *p*-value < 0.01. **k** Representative GO BP terms enriched in the upregulated genes in LM06p-2. **l** TSNE visualization of LM06, LM06p-1, and LM06p-2 using regulon activities (upper left), and representative transcription factor regulon activities of the three subclones (other panels).

Consistently with a previous study^11^, inter-patient heterogeneity was found in our data after annotating cancer cells into more detailed clusters (Fig. 5a, b; Supplementary Fig. S6a, b). The expression levels of cancer stem cell-associated genes *STAT3*, *SOX2*, and *MYC*, varied among cancer cell clusters (Fig. 5c). Non-negative matrix factorization (NMF) analysis revealed eight meta-programs with different characteristics (Fig. 5d; Materials and methods). In particular, meta-program P3, characterized by complement activation and myeloid cell recruitment, was the most commonly shared meta-program among patients (Fig. 5e, f; Supplementary Fig. S6c and Table S5), aligning with the upregulated complement signals observed in LM cancer cells in mice^16^. Chemokine CXCL2, known for recruiting myeloid cells, was expressed in most cancer cells (Supplementary Fig. S6d). These findings align with our observations that myeloid cells dominate the tumor-infiltrating immune cell populations in CNSm. We further applied these meta-programs to MET500 dataset, which includes multiple metastatic tissues^38^. As expected, significantly higher scores of meta-programs P3 were found in CNSm (Fig. 5g), suggesting that recruitment of myeloid cells are conserved features in CNSm.

Cancer cells are heterogeneously and usually comprise multiple subclones with different metastatic potential^39^. We then tried to seek metastasis associated subclones among the 4 four paired samples. In the sample of LM06p (the primary tissue of LM06), we identified two subclones, LM06p-1 and LM06p-2, with LM06p-2 sharing a similar CNV pattern with LM06, the metastatic cancer cell clone (Fig. 5h; Supplementary Fig. S6e). Transcriptomic analysis revealed a high similarity between LM06 and LM06p-2 in UMAP space (Fig. 5i), suggesting that metastatic cancer cells in LM06 likely originated from LM06p-2. Upregulated genes in LM06p-2 were highly enriched in ATP metabolic process and oxidative phosphorylation compared to LM06p-1 (Fig. 5j, k; Supplementary Table S6), consistent with previous findings in breast cancer CNSm^40^. Regulon analysis using SCNEIC^41^ revealed a distinctive regulation network between LM06p-1 and LM06p-2 (Fig. 5l; Supplementary Fig. S6f). As expected, *MYC* regulon activity was higher in LM06 and LM06p-2, consistent with MYC’s role in regulating energy metabolism^42^. In line with our findings, previous studies showed higher MYC amplification in CNSm of lung cancer compared to primary lung cancer^43^. Moreover, *IRF1* and *E2F6*, two metastasis-associated genes^44,45^, also exhibited higher regulon activities in LM06 and LM06p-2 compared to LM06p-1. Our data suggest that LM cancer cells may originate from a subclone of primary NSCLC cells characterized by hyperactive ATP-mediated metabolism and enhanced transcription factor activities promoting metastases. This single-patient study uncovers potential transcriptional signatures of metastatic cancer cells in LM. However, broader validation is required to assess their prevalence and clinical relevance.

### Pro-angiogenic endothelial subtype Endo2 correlated with cancer cell proliferation in BM

**Fig. 6:**
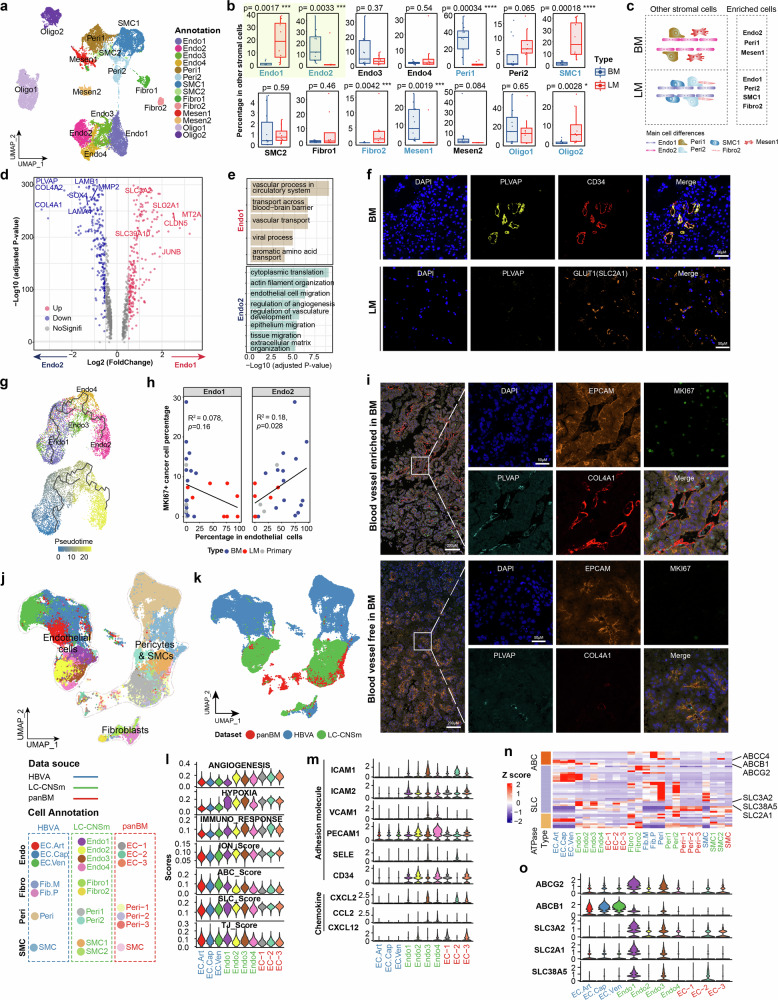
BBB cells were reshaped in CNSm. **a** UMAP visualization of other stromal cells. **b** Cell percentage of other stromal cells between BM and LM. Main cell differences highlighted with yellow transparent background. **c** Schematic diagram of the difference in other stromal cells between BM and LM. **d** Differential gene expression analysis between Endo1 and Endo2. **e** Representative GO Biological Processes gene sets enriched in Endo1 (upper panel) and Endo2 (lower panel). **f** mIHC staining of indicated genes to validate differential features of endothelial cells in BM and LM. **g** Inferred cell trajectory of endothelial cells, colored by endothelial cells (upper panel) and pseudotime (lower panel). **h** Correlation between the percentage of *MKI67* positive cells in cancer cells and endothelial cell subsets (Endo1 and Endo2) in endothelial cells. Samples were colored by tissue types. **i** mIHC staining of indicated genes to show the spatial relationship between blood vessels with angiogenesis features in BM. **j**, **k** UMAP visualization of blood-brain barrier (BBB) associated cells from HBVA, panBM, and our LC-CNSm dataset, colored by cell annotation (**j**) and datasets (**k**). The lower legend depicts the data sources and cell types of each cell cluster. The colors of cell clusters indicated their data sources. HBVA, vascular cells from normal samples; panBM, vascular cells from pan-brain parenchymal metastasis patients, LC-CNSm, our dataset. EC endothelial cells, EC.Art arterial endothelial cells, EC.Cap capillary endothelial cells, EC.Ven venous endothelial cells, Fib fibroblasts, Fib.M matrix associated fibroblasts, Fib.P para-vascular fibroblasts, Peri pericytes, SMC smooth muscle cells. **l** The selective scores of endothelial cells, showing different functional patterns in normal and CNSm conditions, ION ion transporters, ABC ATP-binding cassette transporters, SLC solute carrier transporters, TJ tight junction. **m** Gene expression values of adhesion molecules and chemokines in endothelial cells of 3 datasets. **n** Heatmap of gene expression for differentially expressed transporters in BBB cells. **o** Expression patterns of *ABCG2*, *ABCB1*, and solute carriers, *SLC3A2*, *SLC2A1*, and *SLC38A5* in endothelial cells among three BBB datasets.

Stromal cells are involved in extracellular matrix generation, immune regulation, vascular remodeling and angiogenesis^46^. We thus compared stromal cells between BM and LM, and found substantially difference in cell compositions (Fig. 6a, b; Supplementary Fig. S7a). In specific, endothelial subtype Endo1 cells were more abundant in LM, whereas Endo2 cells were significantly enriched in BM (Fig. 6b, c). Among four subtypes of endothelial cells (ECs), Endo2 exhibited the highest angiogenic capacity and upregulated expression of extracellular matrix-related genes (*COL4A1/2, LAMB1/4*). In contrast, Endo1 had the lowest angiogenic potentials and highly expressed with solute carriers (*SLC3A2*, *SLC2A1*) and the MT family (*MT1E/F/M/X* and *MT2A*) genes (Fig. 6d, e; Supplementary Fig. S7b and Table S7). mIHC stain further validated different phenotypes of endothelial cells between BM and LM (Fig. 6f). We found that the angiogenesis associated genes PLVAP and CD34 were highly expressed in the vasculature of BM, whereas LM lacked PLVAP expression but exhibited high expression of SLC2A1 (encoding GLUT1). Moreover, pseudotime cell trajectory analysis corroborated the differences between Endo1 and Endo2 for these two subtypes located at opposite terminal sides of the UMAP space, with pro-vascular development genes (*PLVAP*, *COL4A1* and *COL4A2*) highly expressed in Endo2 (Fig. 6g; Supplementary Fig. S7c, d). Interestingly, the cancer cells proliferation marker MKI67^47^ correlated well with the proportion of Endo2 but inversely with Endo1 (Fig. 6h; Supplementary Fig. S7e, f). In line with the correlation analysis, blood vessel enriched zone of BM had substantial MKI67 positive cancer cells, but reduced in blood vessel-free zone (Fig. 6i; Supplementary Fig. S7g). This finding may partially explain that Endo2-based angiogenesis is associated with tumor growth and favorable treatment response to Bevacizumab and radiotherapy in BM compared to LM.

### Comparative analysis of BBB in CNSm

Since the BBB is vital for the maintenance of intracranial homeostasis and drug transport^48^, we next compared transcriptional differences of BBB cells utilizing our lung cancer CNSm dataset (LC-CNSm) with public datasets, including a pan-cancer brain metastases dataset (panBM)^11^ as well as a normal human cerebrovascular dataset (HBVA)^49^. BBB cells from CNSm datasets were apparently distinct from those under healthy conditions (Fig. 6j, k; Supplementary Fig. S8a). BBB cells from panBM and LC-CNSm also exhibited high activity scores for immune response and hypoxia, with a lower score for ATP-binding cassette (ABC) transporters (Fig. 6l; Supplementary Fig. S8b). Interestingly, endothelial cells from both LC-CNSm and panBM expressed higher levels of the adhesion molecules (*ICAM1/2*, *SELE*, and *CD34*) and chemokines (*CXCL2* and *CCL2*) (Fig. 6m; Supplementary Fig. S8c), attributing to the recruitment of myeloid cells but rarely T cells. ECM-associated genes were widely expressed in BBB cells, probably promoting collagen and laminin-associated cell-cell interactions that favoring a metastatic environment (Supplementary Fig. S8d, e). Genes in the GTPase activity, cell junctions, and cell-matrix adhesion-related pathways were enriched in ECs, with pericytes and SMCs showing features of cell adhesion and cell junction assembly (Supplementary Fig. S8f and Table S8), indicating impaired vascular integrity and barrier function in CNSm. Furthermore, multi-drug-resistant gene *ABCG2*^50^, as well as solute carriers *SLC2A1*^51,52^, amino acid transporters *SLC38A5* and *SLC3A2*^53–55^ were highly upregulated in Endo1 (Fig. 6n, o). Considering that Endo1 was the major EC subtype in LM, it may partially explain its resistance to intrathecal chemotherapy, tyrosine kinase inhibitors (TKIs) and PD(L)-1 blockades. Collectively, our research demonstrates significant transcriptional alterations in BBB cells, including changes in transporter expression, extracellular matrix production, chemokine secretion, and cell-cell interactions.

Parenchymal and leptomeningeal metastases arise in brain regions with distinct anatomical characteristics, including differences in cerebrovascular endothelial and fibroblast populations^49^. This naturally raises the question of whether the observed differences in the TME are intrinsic to the underlying tissue architecture vs shaped by metastatic tumor growth. We integrated publicly available data from normal human brain parenchyma (BP) and meninges (Men) from previously published studies with our data (Supplementary Fig. S9a−c)^56,57^. Our comparison based on main cell types revealed normal brain parenchyma (BP) contains very few immune or other stromal cells (Supplementary Fig. S9d, e). These observations suggest that the distinct TMEs we identified in BM and LM are primarily shaped by the metastatic process itself, rather than being determined by the pre-existing anatomical architecture. Further fine clustering of macrophages and endothelial cells provided additional evidence supporting the conclusions stated above (Supplementary Fig. S9f−m). These comparative analyses indicate that the differences we identified between BM and LM are driven primarily by metastasis-specific transcriptional programs (Supplementary Fig. S10a).

## Discussion

The current understanding of the TME in LM in situ from lung cancer is limited. This study provides a comprehensive atlas of LM derived from NCCLC, based on directly sampled solid human leptomeningeal metastatic tissue. This atlas is crucial for understanding the adhesive components of the LM TME. We characterized the TMEs in LM and BM, revealing that the LM of lung cancer is a “cold tumor”, featuring minimal plasma cells, dendritic cells, pro-inflammatory macrophages, and CD8^+^ T effector cells, along with increased SPP1 positive myeloid cells. Of note, SPP1 positive myeloid cells are known to be anti-inflammatory and pro-tumor in multiple cancers^33,58,59^. More interestingly, overexpression of SPP1 is associated with poor outcomes in ALK fusion lung cancer patients^60^. Further in vitro experiments showed that targeting the SPP1-CD44 axis restored T cell function, and anti-SPP1 treatment significantly reduced tumor burden, either alone or in combination with anti-PD1 therapy in mouse models^59^. These findings suggest that targeting SPP1^+^ myeloid cells in LM may potentiate the efficacy of immunotherapies like anti-PD1.

On the other hand, BM was infiltrated with more pro-inflammatory myeloid cells such as Macro_CXCL9, CD4T_CXCL13, B cells and CD8^+^ T effectors, which may contribute to improved survival following surgery and targeted therapy. Tumor-infiltration of CXCL9^+^ macrophages and B cells correlates with a better response to immune checkpoint blockades (ICBs) ^30,61,62^. Interestingly, we observed TLS-like formations filled with CXCL13^+^CD4^+^ T cells, CD8^+^ T cells, and B cells in some BM cases, which are associated with enhanced responses to Osimertinib in BM^63,64^. Large-scale clinical analysis also revealed that TLS which characterized by HE and CD3/CD20 staining was an independent prognostic factor for improved response to immunotherapy in patients with solid tumors^65^. This suggests that CD4T_CXCL13, B cells, CXCL9^+^ macrophages, and CD8^+^ T cells in the BM TME may collaborate in response to treatments, resulting in better outcomes. Previous studies have demonstrated that CXCL13^+^ T cells exhibit an exhausted phenotype characterized by elevated exhaustion markers and reduced effector cytokine production, yet retain strong tumor-reactive capacity^66^. Recent single-cell meta-analyses have further corroborated the association between CXCL13^+^ T cells and response to ICB therapy^67^. Intriguingly, single-cell data from breast cancer patients undergoing ICB revealed that both CD4^+^CXCL13^+^ and CD8^+^CXCL13^+^ T cell populations possess predictive value for ICB treatment response. While these findings establish CXCL13^+^ T cells as potential biomarkers for ICB response in primary tumors, their role in predicting CNSm response to ICB requires further experimental validation. Another future direction is to investigate how to reinvigorate these exhausted T cells to enhance their tumor-killing capacity. After all, the majority of tumor-infiltrating lymphocytes (TILs) are bystanders rather than being tumor-reactive^68^.

A higher myeloid/T cell ratio in CNSm or primary CNS cancers has been widely reported^25,69,70^. We found that cancer cells, and endothelial cells expressed *CCL2* or *CXCL2*, which recruit macrophages, explaining the dominance of myeloid cells in CNSm. Moreover, *CCL2* enhances vascular permeability and pulmonary metastases^71^, while *CXCL2* promotes chemoresistances and metastases in lung and breast cancers^72^, and is overexpressed in glioblastoma (GBM) to promote tumor progression^73^. The association between the density of myeloid cell infiltration and poor prognosis has become evident, as many studies across multiple cancers have indicated^74^. Our data demonstrated a dysregulated TME in CNSm, remodeled to enhance tumor cell metastases and survival.

Our study also revealed significant differences in stromal cell compositions between BM and LM. BM exhibited enrichment in the pro-angiogenic endothelial cell subtype Endo2, which supports angiogenesis and nutrient supply for tumor growth. This explains the larger tumor mass in BM compared to LM and supports the use of anti-angiogenic therapies like Bevacizumab and radiotherapy in BM patients. A systematic comparison of BBB cells between CNSm and normal conditions revealed significant phenotypic remodeling. Despite the abnormal expression patterns of solute carrier genes, like GLUT1, in cancer cells across various cancer types^75,76^, our study showed that endothelial cells under CNSm conditions also upregulate GLUT1, providing essential nutrients to cancer cells. We also identified significant upregulation of the multi-drug resistance gene *ABCG2* in BBB cells in CNSm, suggesting its potential as a target for improving the effectiveness of chemotherapy, immunotherapy, or TKI therapy. The phenotypic remodeling in BBB cells under pathological conditions includes dysfunction in lymphocyte recruitment, impaired tight junction, and overexpression of the extracellular matrix, highlighting potential therapeutic intervention avenues in the metastatic process. In addition, we attempted to dissect the transcriptional differences among tumor subclones, potential TF regulators during the leptomeningeal metastasis process in single patient. Larger cohorts and experimental validation are required to validate these observations in further studies.

In conclusion, this study represents a pioneering effort to delineate the key TME features of LM in situ and provides a comparative analysis of the TME for LM vs BM. The findings deepen our understanding of CNSm, offering insights into the local metastatic progression and treatment resistance. Importantly, the single-cell dataset generated in this study serves as a valuable resource for future research exploring the TME of LM and BM. While further mechanistic investigations are essential, this work is crucial in unraveling the molecular mechanisms governing CNSm of NSCLC and discovering novel targets for treating this devastating disease.

## Materials and methods

### Human specimens

Human specimens were collected from patients who were pathologically diagnosed with lung cancer central nervous system metastases, including BM and LM. The cohort included 24 patients (Supplementary Table S1) with metastatic lung cancer disseminated to the brain parenchyma (*n* = 16) or leptomeninges (*n* = 8). Tissues from BM were collected via the surgical resection approach. Paired primary lung cancer tissues (*n* = 4) were collected through computerized tomography-guided percutaneous lung puncture. In patients diagnosed with LM with refractory intracranial hypertension, ventriculoperitoneal shunt and Ommaya reservoir were implanted to relieve high intracranial pressure, and the LM tissues were collected intraoperatively (Supplementary Video S1). This study was approved by the Ethics Committee of Nanfang Hospital, Southern Medical University (NFEC-202105-K12), and informed consent was obtained from all patients.

### Tissue dissociation, single cell suspension preparation, and scRNA-seq library preparations of 10x Genomics

To clean up surface tissue fluid, the tissue was washed with PBS buffer (without Ca^2+^, Mg^2+^) 3 times after being collected by needle biopsy or surgery. Then the sample was immediately placed in a MACS® tissue storage solution (Miltenyi, Germany) at 2−8 °C. The samples were washed with Hank’s balanced salt solution (HBSS) 3 times before dissociation and minced into 1−2 mm pieces. The tissue pieces were digested in 2 mL Tissue Dissociation Solution, the solution containing collagenase (1−2 mg/mL) + DNase I (50 μg/mL) + PBS (with Ca²⁺/Mg²⁺) to prepare the cell suspension. Following digestion, a 40-micron sterile strainer (Corning) was used to separate cells from cell debris and other impurities. The cells were centrifuged at 1000 rpm and 4 °C for 5 min, and then the precipitated cell was resuspended in 1 mL PBS (HyClone). Cell suspensions were counted with a TC20 automated cell counter (Bio-Rad) to determine cell concentration and viability.

The concentration of the single cell suspension was adjusted to 1 × 10^3^ cells/μL in PBS. The single cell suspension was then loaded onto a GEM Chip (10x Genomics, USA). For expression only samples, scRNA-seq libraries were constructed according to the manufacturer’s protocols of Chromium Next GEM Single Cell 3ʹ Reagent Kits v2 (10x Genomics, USA). Briefly, the single cell suspension and Gel Beads were loaded onto the microchip to partition single cells into GEM. Immediately following GEM generation, the Gel Bead was dissolved, primers were released, and any co-partitioned cell was lysed. Incubation of the GEMs produced bar-coded, full-length cDNA from poly-adenylated mRNA. Then the GEMs were broken, and pooled fractions were recovered and used for subsequent cDNA amplification and library construction. After size selection and purification, the scRNA-seq libraries were sequenced on an Illumina NovaSeq 6000 instrument with 150-bp paired-end reads.

For sample with expression and TCR information, single-cell transcriptome and immune repertoire libraries were generated using the Chromium Single Cell 5’ Library & Gel Bead Kit v2 (10x Genomics) according to the manufacturer’s instructions. Cell capture, barcoding, and reverse transcription were carried out on the Chromium Controller. Following cDNA amplification, a portion of the amplified product was used for gene expression library construction, while the remaining material was allocated for T cell receptor (TCR) and B cell receptor (BCR) enrichment using Chromium Single Cell V(D)J Enrichment Kit (10x Genomics). Libraries were sequenced on an Illumina NovaSeq 6000 platform.

### Tissue dissociation, single cell suspension preparation, and single cell RNAseq library preparations of Singleron

Fresh tissues were stored in the GEXSCOPE Tissue Preservation Solution (Singleron Biotechnologies, Nanjing, China) at 2−8 °C immediately after being collected by needle biopsy or surgery. The samples were washed with Hank’s balanced salt solution (HBSS) 3 times before dissociation and minced into 1−2 mm pieces. The tissue pieces were digested in 2 mL GEXSCOPE^TM^ Tissue Dissociation Solution (Singleron Biotechnologies) at 37 °C for 15 min with continuous agitation. Following digestion, a 40-micron sterile strainer (Corning) was used to separate cells from cell debris and other impurities. The cells were centrifuged at 1000 rpm and 4 °C for 5 min, and then the precipitated cell was resuspended in 1 mL PBS (HyClone). Cell suspensions were counted with a TC20 automated cell counter (Bio-Rad) to determine cell concentration and viability.

The concentration of the single cell suspension was adjusted to 1 × 10^3^ cells/μL in PBS. The single cell suspension was then loaded onto a microfluidic chip (Singleron Matrix^®^, Singleron Biotechnologies), and scRNA-seq libraries were constructed according to the manufacturer’s instructions (GEXSCOPE^®^kit, Singleron Biotechnologies). Briefly, a single cell suspension was loaded onto the microchip to partition single cells into individual millipores on the chip. Cell barcoding beads were then loaded into the microchip and mixed with the cell in the millipores. Afterward, 100 μL of single cell lysis buffer was added to the chip to lyse cells and capture mRNAs at room temperature for 20 min. The beads, together with the captured mRNAs, were flushed out of the microchip and used for subsequent reverse transcription, cDNA amplification, and library construction. After size selection and purification, the scRNA-seq libraries were sequenced on an Illumina NovaSeq 6000 instrument with 150-bp paired-end reads.

### Sample collection and preparation for Visium HD (FFPE)

The RNA quality of FFPE tissue blocks was evaluated by calculating DV200 of RNA extracted from FFPE tissue sections following the Qiagen RNeasy FFPE Kit protocol. 5 μm sections were placed on the Sigma-Aldrich Poly Prep Slide following Visium HD Spatial Gene Expression Protocols for FFPE-Tissue Preparation Guide (10x Genomics, CG000684 Rev A). After overnight drying, slides were incubated at 60 °C for 2 h. Deparaffinization was then performed following Visium HD Spatial Gene Expression for FFPE — Deparaffinization, Decrosslinking, Immunofluorescence Staining & Imaging Protocol. Sections were stained with hematoxylin and eosin and imaged at 20x magnification using the brightfield imaging setting on a Leica Aperio Versa8 whole-slide scanner. After that, decrosslinking was performed immediately for H&E stained sections. Next, human whole transcriptome probe panels were then added to the tissue. After these probe pairs hybridized to their target genes and ligated to one another, slides were placed on Visium HD instrument for RNase treatment and permeabilization. The ligated probes were then hybridized to the spatially barcoded oligonucleotides on the Capture Area. Spatial Transcriptomics libraries were generated from the probes and sequenced on the Illumina system.

### Multi-channel immunohistochemistry staining

Multiplexed immunohistochemistry (mIHC) was performed by staining 4-μm-thick formalin-fixed, paraffin-embedded whole tissue sections with standard, primary antibodies sequentially and paired with a TSA 7-color kit (abs50015-100T, Absinbio, Shanghai) followed by staining with DAPI. For example, deparaffinized slides were incubated with anti-CD20 antibody (#abs171788, ABSIN) for 30 min and then treated with anti-rabbit/mouse horseradish peroxidase-conjugated (HRP) secondary antibody (abs50015-02, Absinbio, Shanghai) for 10 min. Next, labeling was developed for a strictly observed 10 min, using TSA 520 per the manufacturer’s instructions. Slides were washed in TBST buffer and then transferred to preheated citrate solution (90 °C) and heat-treated using a microwave set at 20% of maximum power for 15 min. Slides were cooled in the same solution to room temperature. Between all steps, the slides were washed with Tris buffer. The same process was repeated for more antibodies/fluorescent dyes until all targets completed. Each slide was then treated with 2 drops of DAPI (abs47047616, Absinbio, Shanghai), washed in distilled water, and manually coverslipped. Slides were air-dried, and pictures were taken with Pannoramic MIDI II (3DHISTECH). Antibodies used in the study are: CD4 (CST 48274, 1:200); CD8 (CST 70306, 1:200); CD20 (abs171788, 1:500); CXCL13 (ab272874, 1:200); PD1 (CST 86163, 1:200); LAG3 (CST 15372, 1:200); CD68 (ABSIN 171440, 1:200); CCL3 (ABCAM 259372,1:1000); SPP1 (ABCAM 214050, 1:2000); CXCL9 (ABCAM 290643, 1:100); PLVAP (CST 38238, 1:200); GLUT1 (ABCAM 115730, 1:300); COL4A1 (ABCAM 6311, 1:400); CD34 (ABCAM 8536, 1:100); EPCAM (ABSIN 171606, 1:500); Ki-67 (STARTER S0B2332, 1:200). To characterize the exhaustion state of CD8^+^ T cells, a panel consisting of CD8, PD1, and LAG3 was used. To detect diverse lymphocyte populations within the TME, a separate panel including CD20, CD4, CD8, and CXCL13 was applied. Macrophage heterogeneity was investigated using a panel with CD68, CCL3, SPP1, and CXCL9. These three panels were applied to sections from both BM and LM tissues. To investigate the distinct endothelial subtypes, sections from BM were stained for the angiogenesis-associated markers PLVAP and CD34 to identify Endo2, while LM sections were stained for the Endo1-specific marker GLUT1 (encoded by SLC2A1). PLVAP staining was also performed on LM sections to assess for angiogenic features. Finally, to explore the relationship between the Endo2 subtype and cancer cell proliferation, a separate panel consisting of PLVAP, COL4A1, EPCAM, and MKI67 was utilized. All sections were counterstained with DAPI.

### scRNA-seq data processing

For sequences obtained from samples prepared with 10x Genomics samples, Cell Ranger software (v.6.0.2) was downloaded from 10x Genomics (https://support.10xgenomics.com/single-cell-gene-expression/software/downloads/latest) and used to process raw data, filter low-quality data, align reads to the hg38 human reference genome, and summarize unique molecular identifier (UMI) counts. For samples sequenced by Singleron, CeleScope (version 1.11.1) was run to process raw data via a similar procedure. Seurat (version 4.0.1)^77^ was used for most single-cell data downstream analyses. Before the raw UMI counts matrix was loaded into Seurat, genes detected in fewer than 3 cells, and cells with fewer than 200 genes were removed. Doublets were removed by DoubletFinder (version 2.0.3)^78^. Furthermore, we calculated the metrics of each cell, and cells with more than 15% mitochondrial gene counts, fewer than 500 genes, and more than 5000 UMI counts were removed. After all single samples were merged into one Seurat object, the SCTransform function was applied to the data with the regress variables “percent.mt”, “S.Score”, and “G2M.Score”.

### Dimension reduction and clustering for scRNA-seq data

The top 3000 highly variable genes (HVGs) were automatically detected by the SCTransform function. PCA was performed on the HVGs, and the top 50 principal components were used in downstream analysis. To remove batch effects from different droplet-based sequencing techniques, Harmony (version 0.1.0)^79^ was applied with setting sequencing technology as batch variable. The top 30 components from Harmony were selected to execute the RunUMAP function with all other parameters set to their default values in Seurat, generating a non-linear dimension reduction projection using Uniform Manifold Approximation and Projection (UMAP) for visualization. A K nearest neighbor (KNN) graph was generated by FindNeighbors using the top 30 Harmony components with all other parameters set to their default values, and clusters were classified by the FindClusters function with a manually selected resolution from 0.5 to 1.5. To obtain better annotation results, we undertook 2 rounds of cell annotation. For the first round of annotation, a resolution of 0.8 was chosen to classify clusters, and canonical cell markers were used to annotate cell clusters into myeloid cells, T/NK cells, B cells, cancer cells, and other stromal cells. The marker list can be found in supplementary tables. After the first round of annotation, we obtained the main cell types. Then a second round of annotation was run based on each major cell type, starting from the filtered cell expression count matrix. Notably, the second round of annotation was annotated based on the signature genes of each cluster.

To obtain top signature genes of each cell clusters, FindAllMarkers function in Seurat with setting only.pos = TRUE, min.pct = 0.25, logfc.threshold = 0.25 was performed. To detect differential expression genes (DEGs) in specific cell clusters, we ran FindMarkers function in Seurat package with setting only.pos = F, min.pct = 0.25, logfc.threshold = 0.25. DEGs are defined as the absolute values of average log2 fold changes ≥ 0.5 and adjust *p*-values ≤ 0.05. Enrichment analyses were all performed by clusterProfiler (v.4.2.0)^80^ with default settings.

### Spatial transcriptomics (ST) data preprocessing

For ST data, Space Ranger software (v.3.1.3) were downloaded from 10x Genomics (https://www.10xgenomics.com/support/cn/software/space-ranger/downloads) and used to process raw data, align reads to the hg38 human reference genome, and summarize UMI counts using default parameters. The annotation file version was refdata-gex-GRCh38-2020-A and can be downloaded from the URL (https://www.10xgenomics.com/support/cn/software/space-ranger/downloads#reference-downloads). Visium HD CytAssist image alignment was performed by Loupe Browser software (v.8.1.2). As recommended by 10x Genomics, we used the expression matrix at square_008um resolution for downstream analyses. The R package Seurat (v.5.2.1) was applied to perform following analysis for Seurat v5 having better support for ST data. We calculated the metrics of each cell, and cells with fewer than 10 UMI counts per 8 μm bin were removed. The expression data of left cells were log normalized and then scaled, using NormalizeData, ScaleData function in sequence. Then we generated a sketch assay containing 50,000 cells and performed PCA, UMAP reduction and clustering in the sketch assay. The clustered sketch data were then projected to its full assay. Classical markers were used to annotate the main cell types of ST data.

### Trajectory analysis for specific cell subtypes

To investigate the continuous cellular differentiation and state transition processes, we performed pseudotime trajectory analysis using the R package Monocle3 (v.1.3.4)^81^. A cell_data_set (CDS) object was constructed from the pre-processed single-cell data, which included the normalized expression data, cell metadata. The analysis workflow was performed as the tutorial of the Monocle3 framework, including dimension reduction using Principal Component Analysis (PCA), followed by UMAP algorithm. Before get UMAP space, the align_cds function was also run to correct batch effect. A principal graph representing the potential cellular trajectories was learned using the learn_graph() function, and after that, the order_cells() function was used to calculate the pseudotime value for each cell.

### TCR/BCR sequence assembly and analysis

Cell Ranger software (v.6.0.2) was used to align TCR/BCR reads to human reference genome hg38 and assemble TCR/BCR sequences. The preliminary TCR/BCR sequences were filtered to maintain high-confidence, full-length, productive sequences. Downstream analysis of TCR/BCR was done using mainly scRepertoire (1.0.0)^82^. A unique clone was defined by “gene+nt” in scRepertoire. Clone expansion definition: Single (x ≤ 1), Small (1 < x ≤ 5), Medium (5 < x ≤ 20), Large (20 < x ≤ 100), Hyperexpanded (100 < x ≤ 500); x means detected cells per clone. Clone abundance of T cell clusters was calculated by ratios of each clone. Criteria: Rare (0 < X ≤ 1e − 04), Small (1e − 04 < X ≤ 0.001), Medium (0.001 < X ≤ 0.01), Large (0.01 < X ≤ 0.1), Hyperexpanded (0.1 < X ≤ 1), where X means the ratio of a clone in all clones.

### CNV analysis

CNV was estimated by the R package inferCNV (version 1.12.0)^83^. An annotation file was generated from GTF using Cell Ranger. SCT counts were used to feed inferCNV with the main cell types as annotations. Notably, all cells without epithelial cell marker gene expression were regarded as reference cells. InferCNV was run on each sample with the following parameters: cutoff = 0.1, cluster_by_groups = TRUE, denoise = TRUE, and HMM = F. To create a comparable CNV plot, we re-ran the plot_cnv function with the following parameters: plot_chr_scale = T, x.range = c(0.9,1.1), and x.center = 1. To merge the CNV plot of all samples, we applied ComplexHeatmap (version 2.12.1)^84^ to create a genome-level heatmap.

### Meta-program discovery procedure by NMF analysis

We used the NMF algorithm in the R package nmf (version 0.23.0)^85^ to factorize the scaled and centered expression data on tumor cells in each sample after converting all negative values to zero. For each sample, 5 NMF factors were chosen, and the 50 genes with the highest NMF scores of each NMF factor were regarded as signature genes of NMF programs. To find recurrent programs, which were also called meta-programs, we used all programs from a single sample, calculated the PCC of each 2 programs, calculated the distances of programs with 1 minus the PCC value, and manually selected 8 meta-programs. To dissect the properties of meta-programs, we used the signature genes in each meta-program to enrich the GO biological process (BP). To refine the genes of the meta-program, we counted the meta-program scores using AUCcell^41^ and calculated PCC between meta-program signature genes and AUCcell scores. We selected the top 30 genes most correlated with the meta-program score as the refined signature genes for each meta-program.

### Definition of single cell gene signature scores

All single cell gene signature scores were calculated by R package AUCcell (v 1.12.0)^41^. Cell ranking was calculated by the AUCell_buildRankings function with normalized data, and AUCcell scores were counted by the AUCell_calcAUC function with aucMaxRank equaling the row number of cell ranking * 0.1. Genesets using in T/NK cells were downloaded from the supplementary file of the study^11^. Hallmark datasets were downloaded from the MsigDB database (https://www.gsea-msigdb.org/gsea/msigdb). Genesets to count the functional activity of endothelial cells, including ion channel (ION) scores, tight junction (TJ) scores, ATP-binding cassette transporter (ABC) scores, and solute carrier (SLC) scores, using ‘REACTOME_ION_CHANNEL_TRANSPORT’, ‘KEGG_TIGHT_JUNCTION’, ‘KEGG_ABC_TRANSPORTERS’, and ‘REACTOME_METAL_ION_SLC_TRANSPORTERS’) as reference gene sets.

### Cell ratio of cell types and correlation of immune cells

For immune cells, we counted the cellular proportions of each cluster in all immune cells, including T/NK cells, myeloid cells, and B cells. For other stromal cells, the cellular proportions of each cluster were counted in all other stromal cells, including endothelial cells, smooth muscle cells, pericytes, fibroblasts, and mesenchymal cells. To avoid variance from a low total cell number, samples with major cell types of fewer than 100 were removed. After acquiring these cellular ratio data, we compared cell ratio differences between BM and LM and between primary and metastases samples. *p* values were calculated by Wilcoxon test. To validate the results of our cell proportion-based analysis, the Cacoa (v.0.5.0) tool was applied to our data, with BM set as the reference condition and reference cells automatically detected by the algorithm^24^. The cell ratios of T/NK cells, myeloid cells, and B cells were calculated by PCC to detect the potential connection between immune cells. To calculate similarity of TME between BM, LM, and primary lung cancer, we first counted mean cell ratio of three conditions, and got rank value of each cluster, then Kendall coefficient was calculated.

### Public datasets download and integrative analysis with published datasets

MET500 dataset was downloaded from UCSC Xena data Hubs using R package UCSCXenaTools (version 1.4.8)^86^. No raw count data of the MET500 dataset were found, and FPKM values were used in downstream analysis. To count the signature scores of each sample, the calculate_sig_score function in IOBR was run with the ssGSEA method. The panBM dataset, a pan-cancer BM scRNA-seq dataset, was downloaded from the GEO website (https://www.ncbi.nlm.nih.gov/geo/) with accession number GSE186344^11^. The HBVA dataset, which was a normal human BBB-associated cell dataset, sequenced by a droplet-based single nucleus, was also downloaded from GEO with accession number GSE163577^49^. Gene markers for subtypes of endothelial cells were downloaded from supplementary file of GSE163577^49^.

To further verify the functional roles of our identified macrophage clusters, we attempted to integrate our dataset with a single-cell study on anti-PD-1 therapy in breast cancer^37^. We used the genes from the work. Upregulated genes are the feature genes of macrophages positively correlated with T cell expansion, referred to as E-genes, while downregulated genes are the feature genes of macrophages negatively correlated with T cell expansion, termed NE-genes. These E-genes and NE-genes were fed AddModuleScore function in Seurat to calculate E-scores and NE-scores, visualized by SCP (v.0.5.6) package.

The expression data from HBVA, panBM and our data was integrated with data source as batch variable by Harmony, and then run standard Seurat pipeline. Cell labels were directly retrieved form original annotation, and visualized by UMAP. To compare transcriptional differences of BBB cells between normal and CNSm, we first classified the three datasets (HBVA, panBM and our LC-CNSm) into 4 parts, including HBVA, panBM, LM samples LC-CNSm-LM and BM samples LC-CNSm-BM. Then, each part of all three CNSm parts was performed differential expression (DE) analysis with the normal HBVA dataset in all main BBB cells. To compare functional pathways (including ABC, SLC, ATPase associated transporters) of BBB cells between normal and CNSm, gene lists were downloaded from the website with the URl: (https://www.guidetopharmacology.org/download.jsp).

Parenchymal and leptomeningeal metastases arise in brain regions with distinct anatomical characteristics. To determine whether the observed differences in the TME are intrinsic to the underlying tissue architecture versus shaped by metastatic tumor growth, we integrated publicly available data from normal human brain parenchyma (BP) and meninges (Men) from previously published studies with our data^56,57^. BP data was download from the URL (https://github.com/LieberInstitute/10xPilot_snRNAseq-human). Men data was download from the data sharing platform Zenodo (10.5281/zenodo.4932158). To ensure comparability, we downsampled the data from each of the four sources to 20,000 cells, integrated these data, and annotated main cell types using canonical markers. To further investigate cell composition among the four data source, macrophages and endothelial cells were further re-annotated into detailed clusters. The position of cells in UAMP space, cell number of each main cell type or cell clusters, and feature genes of cell subtypes identified from our former single-cell analysis were compared among BP, Men, BM and LM.

## Supplementary information

Supplementary Video S1: The process of LM tissue acquisition.

Supplementary Table S1

Supplementary Table S2

Supplementary Table S3

Supplementary Table S4

Supplementary Table S5

Supplementary Table S6

Supplementary Table S7

Supplementary Table S8

Supplementary Fig. S1: Cell atlas of CNSm, related to Fig. 1.

Supplementary Fig. S2: Cell characteristics of T/NK cells, related to Fig. 2.

Supplementary Fig. S3: TCR analysis of T cells, related to Fig. 2.

Supplementary Fig. S4: Lymphocyte difference between BM and LM, related to Fig. 3.

Supplementary Fig. S5: Characteristics of myeloid cells, related to Fig. 4.

Supplementary Fig. S6: Characteristics of cancer cells, related to Fig. 5.

Supplementary Fig. S7: Characteristics of stromal cells, related to Fig. 6.

Supplementary Fig. S8: Comparison of normal and CNSm condition BBB cells, related to Fig. 6.

Supplementary Fig. S9: Integration analysis among our data, published normal human brain parenchyma and meninges, related to Fig. 6.

Supplementary Fig. S10: Graphical abstract of the study.

## Data Availability

Data associated with the work has been deposited at NODE (The National Omics Data Encyclopedia) with access number OEP004944 and is accessible to through this link: https://www.biosino.org/node/project/detail/OEP004944 approved by the corresponding author. The analysis procession of the paper has been described in Methods. The analysis scripts for study have uploaded to the Github website (https://github.com/shanshenbing/LC-CNSm). Any additional information required to reanalyze the data reported in this paper is available from the leading contact upon request.
