# Supplementary material for: Immunological and pathological characteristics of brain parenchymal and leptomeningeal metastases from non-small cell lung cancer
Source: Cell Discov. 2025 Aug 29;11:72. doi: 10.1038/s41421-025-00828-7 (PMC12397330; doi:10.1038/s41421-025-00828-7)
Supplement: Supplementary file 10 — Supplementary Fig. S1: Cell atlas of CNSm, related to Fig. 1. [file 41421_2025_828_MOESM10_ESM.pdf]

Supplementary Fig. S1

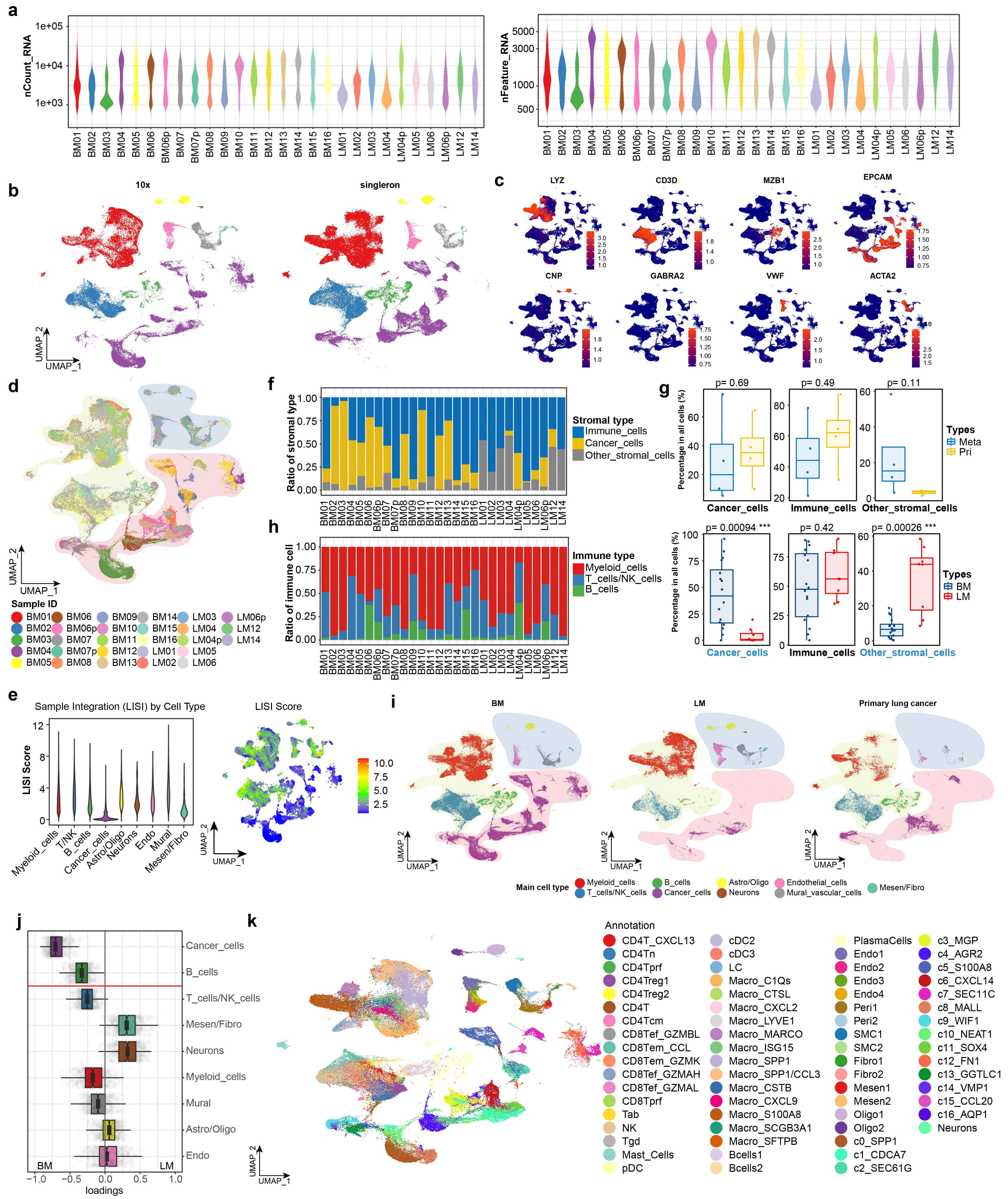

Supplementary Fig. S1: Cell atlas of CNSm, related to Fig. 1.

(**a**) UMI counts of each sample (left panel); gene numbers of each sample (right panel). (**b**) UMAP visualization of samples sequenced by 10x Genomics and Singleron. (**c**) Expression of representative marker genes of main cell types. (**d**) UMAP visualization of main cell types, colored by samples. (**e**) The Local Inverse Simpson's Index (LISI) in each main cell types, visualized by violin plot (left panel), and by UMAP (right panel). (**f**) Stromal cell abundance in each sample. Stromal types were defined as immune cells, cancer cells, and other stromal cells. (**g**) Comparisons of percentages of stromal cell types in all cells between primary and metastatic samples (upper panel), and between BM and LM samples (lower panel). P value was calculated by the Wilcoxon test. (**h**) Immune cell abundance in each sample. Immune cells were including myeloid cells, T/NK cells and B cells. (**i**) UMAP visualization of main cell types in tissue types. (**j**) Main cell type differences between BM and LM, calculated by Cacoa. (**k**) UMAP visualization of all cells, colored by fine annotation.
