# Supplementary material for: Immunological and pathological characteristics of brain parenchymal and leptomeningeal metastases from non-small cell lung cancer
Source: Cell Discov. 2025 Aug 29;11:72. doi: 10.1038/s41421-025-00828-7 (PMC12397330; doi:10.1038/s41421-025-00828-7)
Supplement: Supplementary file 11 — Supplementary Fig. S2: Cell characteristics of T/NK cells, related to Fig. 2. [file 41421_2025_828_MOESM11_ESM.pdf]

Supplementary Fig. S2

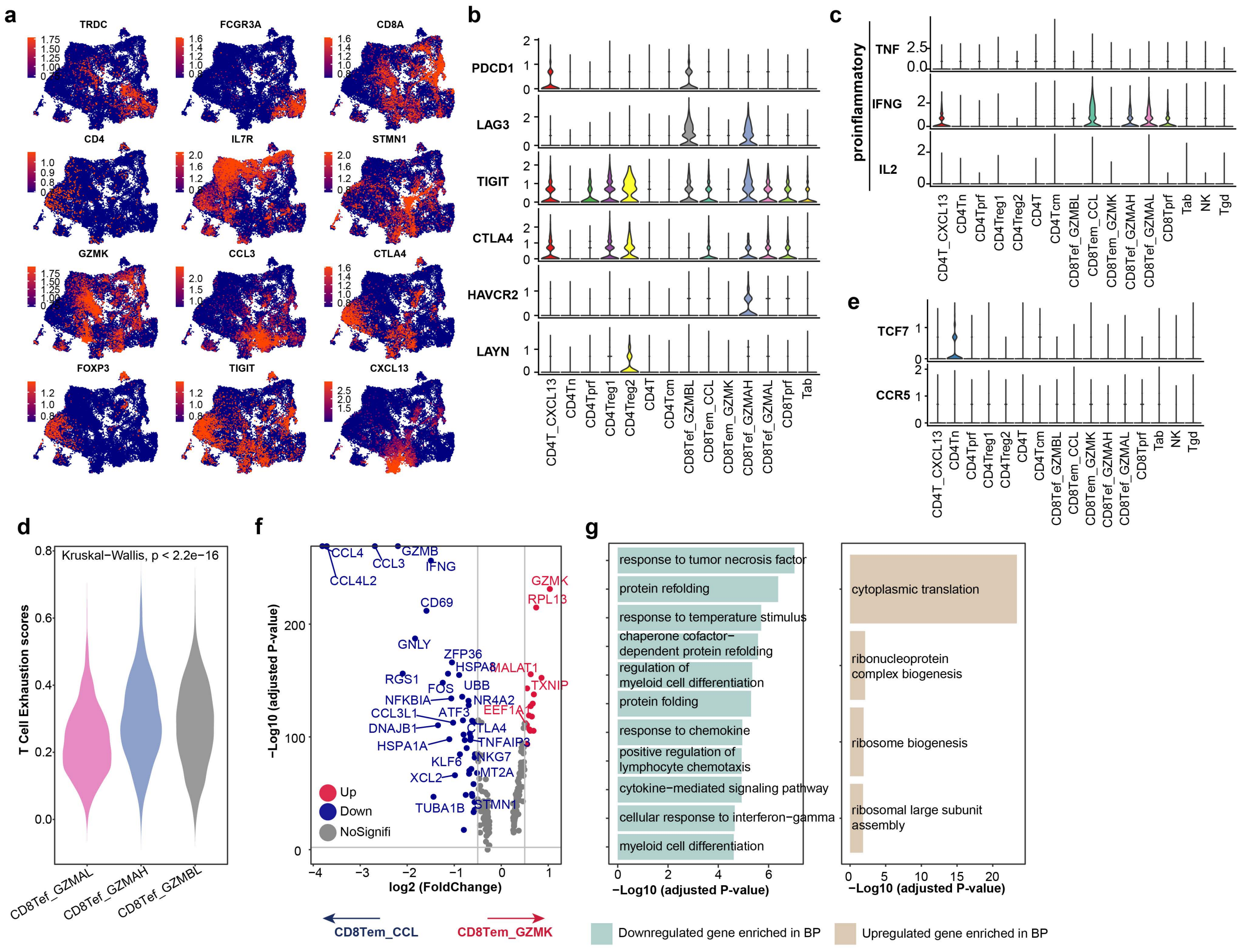

Supplementary Fig. S2: Cell characteristics of T/NK cells, related to Fig. 2.

(**a**) Expression patterns of representative feature genes in T/NK cells. (**b**) Expression of exhaustion gene markers in different cell clusters of T/NK cells. (**c**) Expression patterns of proinflammatory genes in T/NK cells. (**d**) Exhaustion gene scores in three effector T cells. (**e**) Expression pattern of *CCR5* and *TCF7* in T/NK cells. (**f**) Differential expression analysis of CD8Tem_GZMK and CD8Tem_CCL. Criteria: log2 fold change >= 0.5, adjust p value < 0.01, adjust p value method: BH. (**g**) Enriched gene ontology (GO) biological process (BP) terms in 2 CD8+ T memory cells. Left panel: CD8Tem_CCL enriched GO BP terms; Right panel: CD8Tem_GZMK enriched GO BP terms.
