# Supplementary material for: Immunological and pathological characteristics of brain parenchymal and leptomeningeal metastases from non-small cell lung cancer
Source: Cell Discov. 2025 Aug 29;11:72. doi: 10.1038/s41421-025-00828-7 (PMC12397330; doi:10.1038/s41421-025-00828-7)
Supplement: Supplementary file 12 — Supplementary Fig. S3: TCR analysis of T cells, related to Fig. 2. [file 41421_2025_828_MOESM12_ESM.pdf]

Supplementary Fig. S3

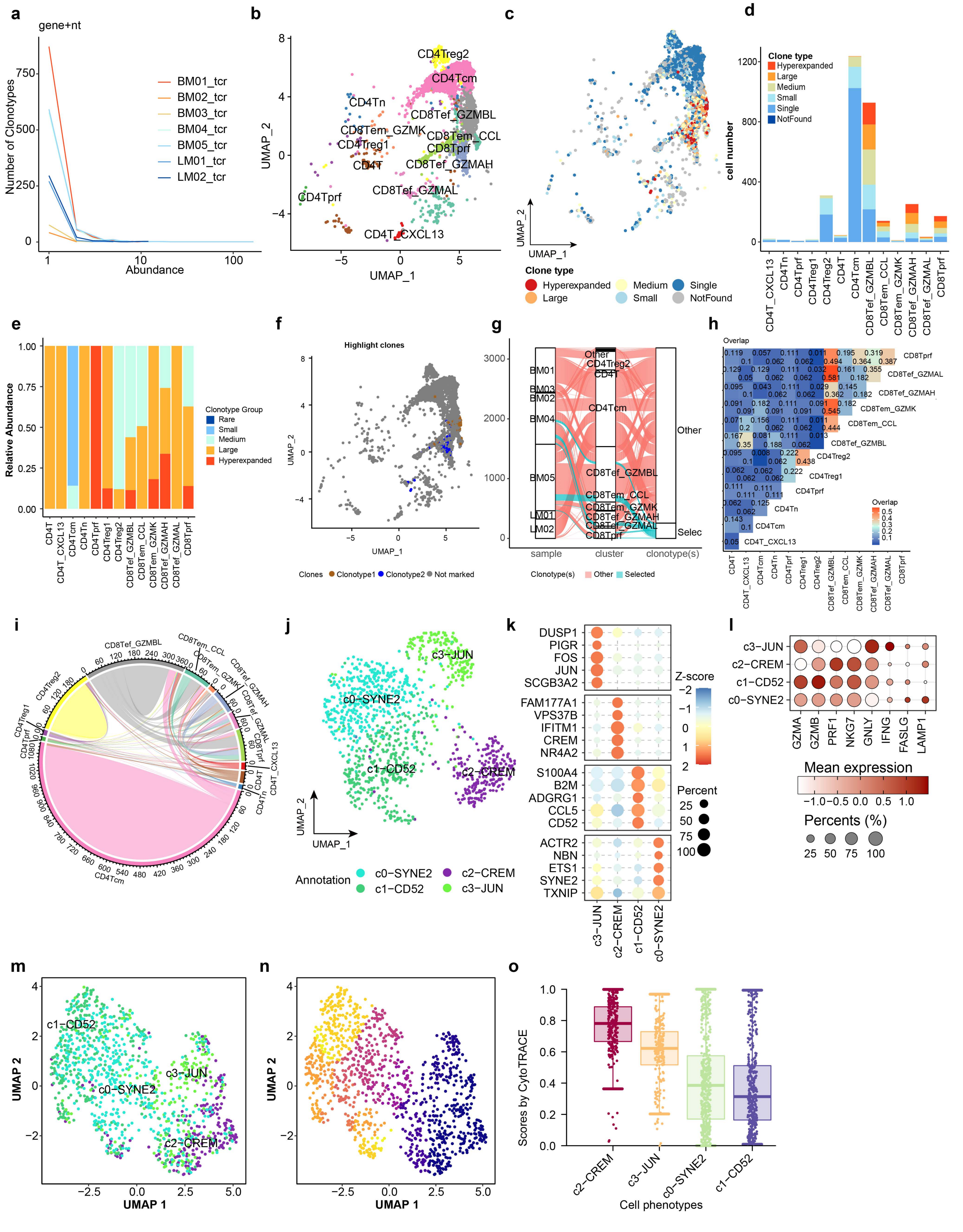

Supplementary Fig. S3: TCR analysis of T cells, related to Fig. 2.

(**a**) Clone abundance in 7 sequenced TCR samples. A clone was defined with a unique VDJ gene and CDR sequence. (**b**) UMAP visualization of scRNA-seq data of only 7 samples with both scTCR-seq and scRNA-seq. (**c**) Clone expansion levels, visualization by UMAP, colored by the degree of clone expanded. (**d**) Clone expansion levels, visualization by bar plot. Clone expansion definition: Single (x<=1), Small (1<x<=5), Medium (5<x<=20), Large (20<x<-100), Hyperexpanded (100<x<=500); x means detected cells per clone. (**e**) Clone abundance of T cell clusters, calculated by ratios of each clone. Criteria: Rare (0 < X <= 1e−04), Small (1e−04 < X <= 0.001), Medium (0.001 < X <= 0.01), Large (0.01 < X <= 0.1), Hyperexpanded (0.1 < X <= 1), where X means the ratio of a clone in all clones. (**f**) Highlighting 2 hyperexpanded clone types. (**g**) Sanky plot showing samples and cell type distribution of highlighted clones. (**h**) TCR overlap ratio in T cell clusters. (**i**) Circular plot depicting the shared TCR of each T cell cluster. The number on the outer layer of the plot represented the clone number of each cluster, while the different sector represented different clusters; the inner strips meant clones, while strips connecting more than one sector represented shared clones. (**j**) UMAP visualization of the sub-clustered NK clusters. NK cells were colored by clusters. (**k**) The expression pattern of top marker genes in each NK cell cluster. (**l**) The expression pattern of cytotoxic genes in NK subtypes. (**m**) The trajectory layout of NK cell transition inferred by Monocle3. Cells were colored by cell clusters. (**n**) The pseudotime of the inferred trajectory of NK cell development. (**o**) The stemness scores estimated by CytoTRACE.Supplementary
