# Supplementary material for: Immunological and pathological characteristics of brain parenchymal and leptomeningeal metastases from non-small cell lung cancer
Source: Cell Discov. 2025 Aug 29;11:72. doi: 10.1038/s41421-025-00828-7 (PMC12397330; doi:10.1038/s41421-025-00828-7)
Supplement: Supplementary file 13 — Supplementary Fig. S4: Lymphocyte difference between BM and LM, related to Fig. 3. [file 41421_2025_828_MOESM13_ESM.pdf]

Supplementary Fig. S4

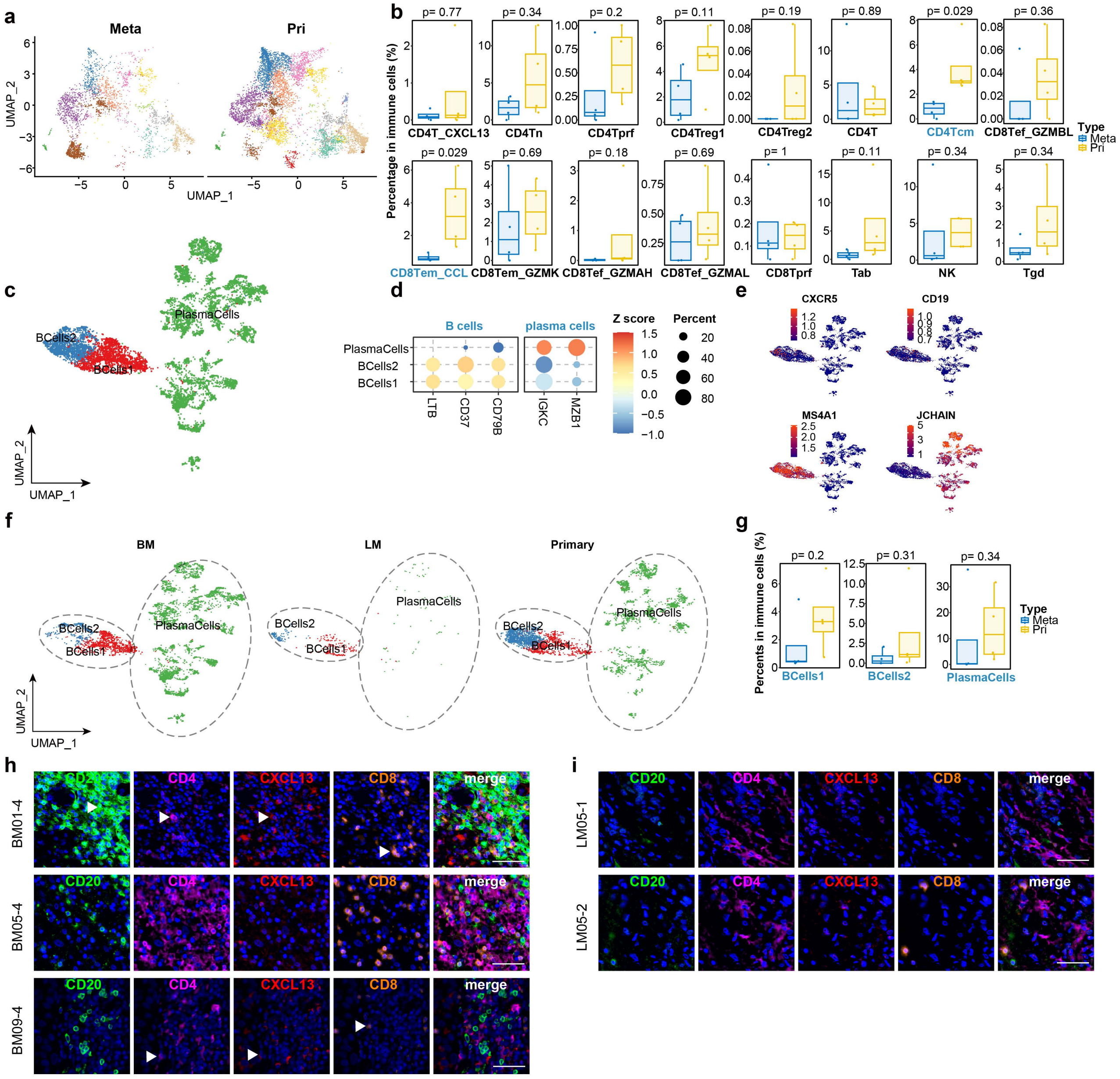

Supplementary Fig. S4: Lymphocyte difference between BM and LM, related to Fig. 3.

(**a**) UMAP visualization of T/NK cells, split by primary and CNSm. Abbreviations: pri, primary cancer; meta, metastasis cancer. (**b**) Cell ratio differences between primary cancer and CNSm. Each dot represented 1 sample. Box middle lines, median values; box limits, upper and lower quartiles; box whiskers, 1.5 × the interquartile range. Significance was calculated by Wilcoxon test. Abbreviations: pri, primary cancer; meta, CNSm. (**c**) UMAP visualization of B cells. B cells were annotated into PlasmaCells, BCells1, and BCells2. (**d**) B cell marker gene dot plot. Gene expression was scaled by columns, and dot size was scaled by the percent of each gene expression in cell types. (**e**) Expression patterns of representative normalized feature genes in B cells. (**f**) UMAP visualization of T/NK cells, split by BM, LM, and primary. Grey dashed circles marked differences in B cells. (**g**) Cell ratio differences between primary cancer and CNSm. (**h**) Selected genes stained by multiple channel IHC in three BM patients. Scale bar: 50 μm. (**i**) Selected genes stained by multiple channel IHC in LM patient. Scale bar: 50 μm.
