# Supplementary material for: Immunological and pathological characteristics of brain parenchymal and leptomeningeal metastases from non-small cell lung cancer
Source: Cell Discov. 2025 Aug 29;11:72. doi: 10.1038/s41421-025-00828-7 (PMC12397330; doi:10.1038/s41421-025-00828-7)
Supplement: Supplementary file 14 — Supplementary Fig. S5: Characteristics of myeloid cells, related to Fig. 4. [file 41421_2025_828_MOESM14_ESM.pdf]

Supplementary Fig. S5

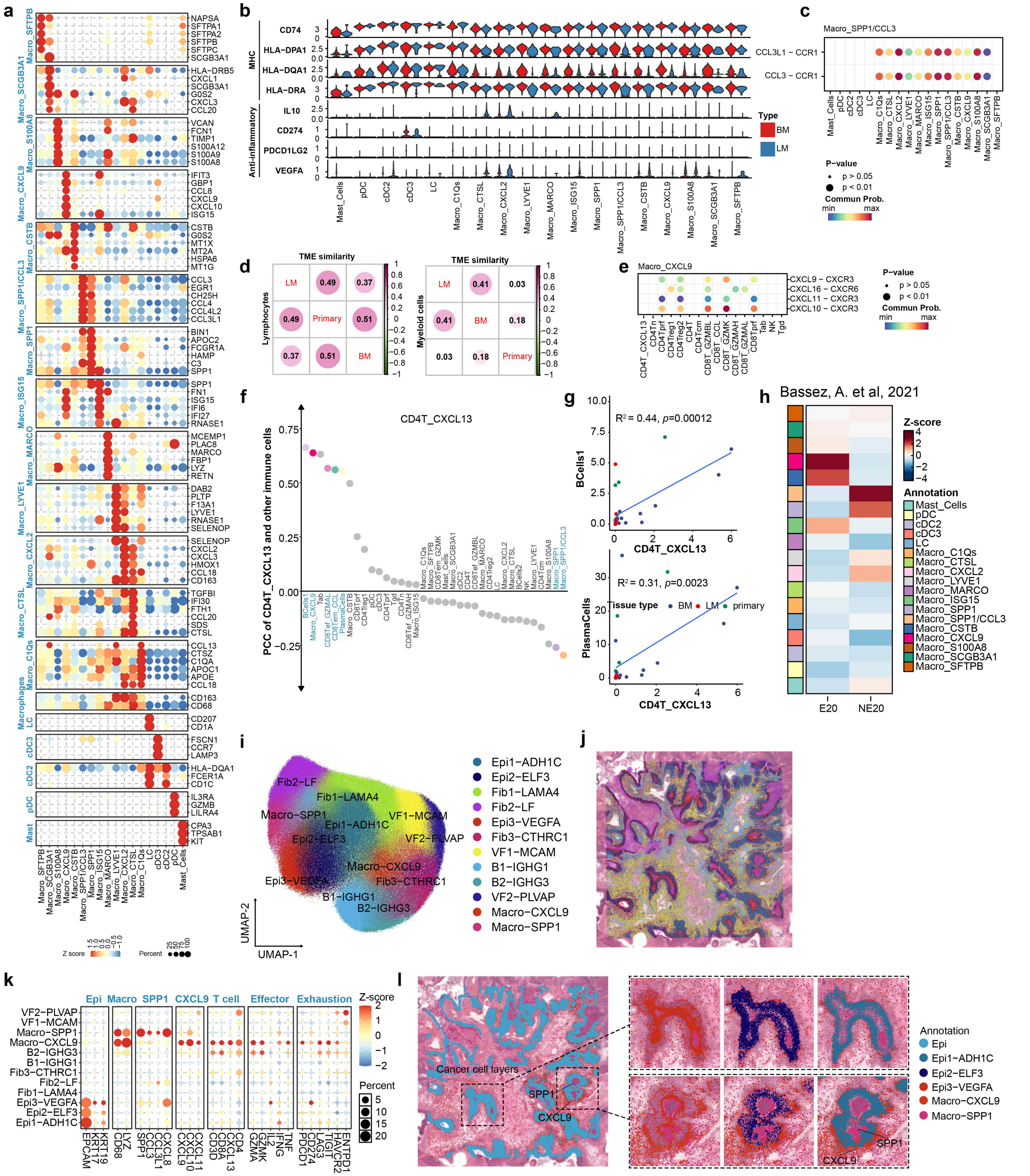

Supplementary Fig. S5: Characteristics of myeloid cells, related to Fig. 4.

(**a**) Myeloid cell signature gene dot plot. Gene expression was scaled by columns, and dot size was scaled by the percent of each gene expression in cell types. Abbreviation: Macro, macrophages; cDC, classical dendritic cells; pDC, plasmacytoid dendritic cells; LC, Langerhans-like cells. (**b**) Expression pattern of anti-inflammatory and antigen presentation-associated genes between BM and LM. (**c**) Cell-cell communication between Macro_SPP1/CCL3 and myeloid cells. Results of cell-cell communication were calculated by Cellchat. (**d**) TME similarity among BM, LM, and primary lung cancer. The clusters were ranked by the mean percentage in lymphocytes (left panel) and myeloid cells (right panel), and similarities were calculated by the Kendall coefficient. The size of the points and the intensity of the color indicate the magnitude of the similarity. (**e**) Cell-cell communication between Macro_CXCL9 and T/NK cells. (**f**) The correlation of the relative abundance between CD4T_CXCL13 and all other immune cell clusters. Representative cell clusters are highlighted. (**g**) The correlation of the percentage of CD4T_CXCL13 and Bcells1 (upper panel) and PlasmaCells (lower panel). Each point represents one sample, and points were colored by tissue type. (**h**) Signature scores of myeloid cells. The feature genes of macrophages are positively correlated with T cell expansion, referred to as E-genes, while the genes are negatively correlated with T cell expansion, termed NE-genes. E-genes and NE-genes are used to calculate E-scores and NE-scores, respectively. (**i**) UAMP visualization of spatial transcriptomics clusters. Abbreviations: Epi, epithelial-derived tumor cells; Fib, fibroblasts; VF, vascular or fibroblast cells; B, B cells; Macro, macrophages. (**j**) The distribution of spatial transcriptomics clusters across the tissue section. (**k**) Feature genes shown differences between Macro_CXCL9 cluster and Macro_SPP1 cluster. (**l**) The spatial distribution of cancer cells and macrophages. The cancer cells can be further classified into different layers by transcriptomics differences (right-upper panel), and spatial organization of macrophages and cancer cells (right-lower panel).
