# Supplementary material for: Immunological and pathological characteristics of brain parenchymal and leptomeningeal metastases from non-small cell lung cancer
Source: Cell Discov. 2025 Aug 29;11:72. doi: 10.1038/s41421-025-00828-7 (PMC12397330; doi:10.1038/s41421-025-00828-7)
Supplement: Supplementary file 15 — Supplementary Fig. S6: Characteristics of cancer cells, related to Fig. 5. [file 41421_2025_828_MOESM15_ESM.pdf]

Supplementary Fig. S6

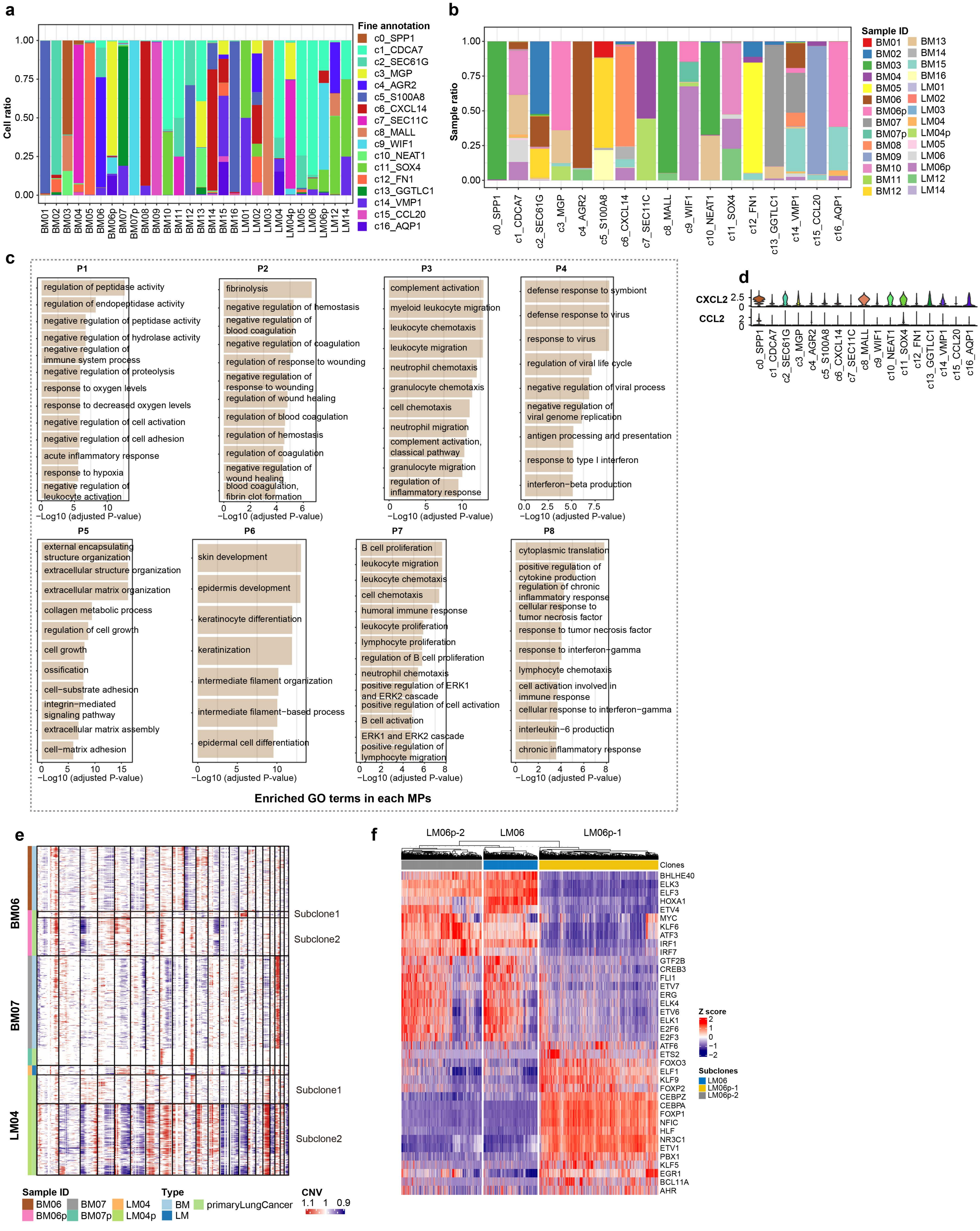

Supplementary Fig. S6: Characteristics of cancer cells, related to Fig. 5.

(**a**) Cancer cell cluster ratios in each sample. (**b**) Sample compositions in each cancer cell cluster. (**c**) Representative enriched GO BP terms of meta-programs. From left to right, from top to bottom, P1 to P8. (**d**) Expression patterns of representative cytokines/chemokines in cancer cells. (**e**) CNV patterns of BM06, BM07, LM04, and primary pairs from top to bottom. Potential subclones of BM06p and LM04p were marked with a black rectangular box. Each row was a cell, and each column was a chromosome. (**f**) Regulon activities of LM06, LM06p-1, and LM06p-2.
