# Supplementary material for: Immunological and pathological characteristics of brain parenchymal and leptomeningeal metastases from non-small cell lung cancer
Source: Cell Discov. 2025 Aug 29;11:72. doi: 10.1038/s41421-025-00828-7 (PMC12397330; doi:10.1038/s41421-025-00828-7)
Supplement: Supplementary file 16 — Supplementary Fig. S7: Characteristics of stromal cells, related to Fig. 6. [file 41421_2025_828_MOESM16_ESM.pdf]

Supplementary Fig. S7

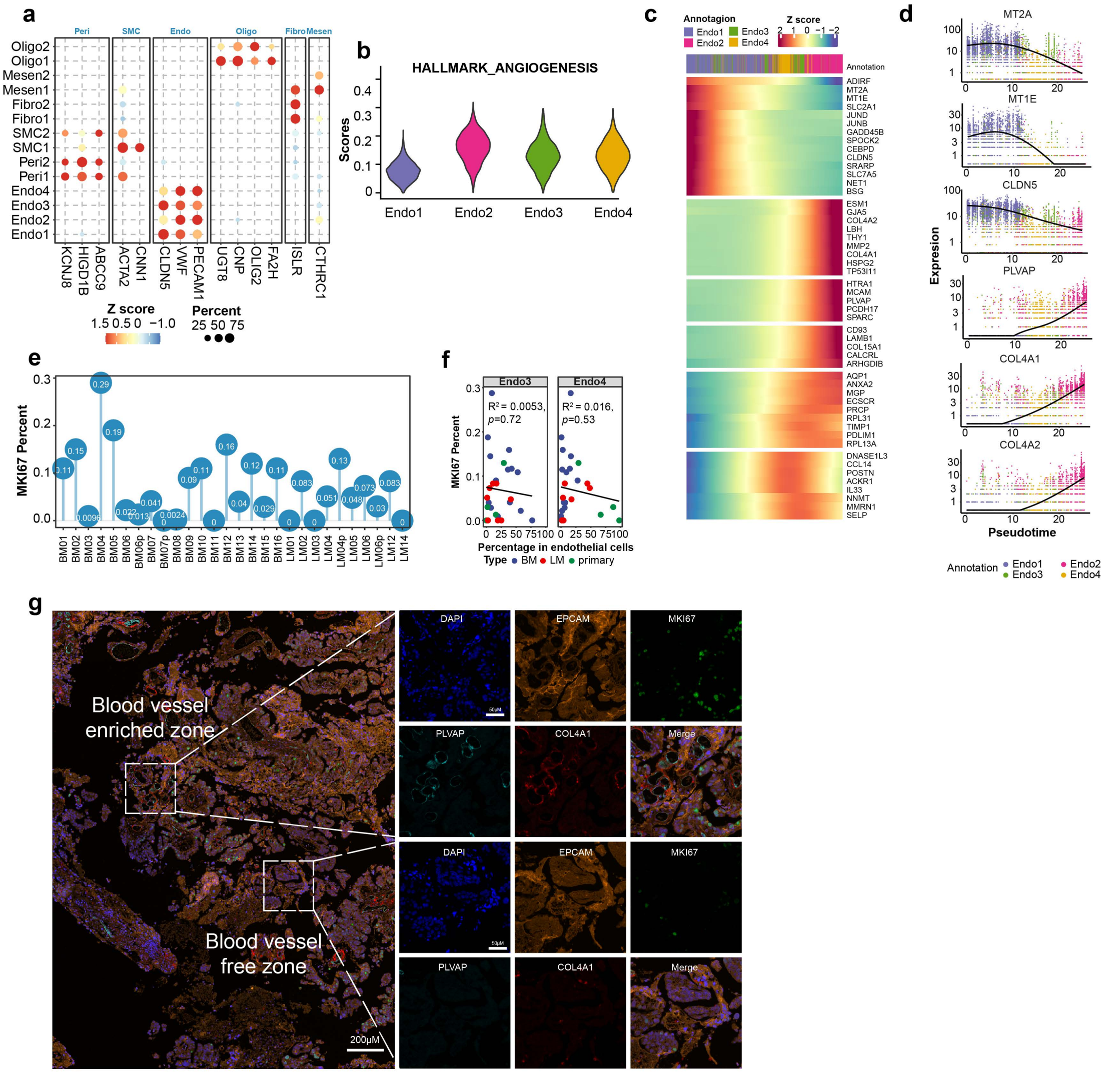

Supplementary Fig. S7: Characteristics of stromal cells, related to Fig. 6.

(**a**) Expression of marker genes in different other stromal cell clusters. Individual gene expression was normalized to overall gene expression, and dot size indicates the percentage of cells expressing the genes in different cell clusters. (**b**) Signature score calculated by AUCcell, visualized by violin plot of endothelial cells. (**c**) Representative gene expression changes along increase of inferred pseudotime. (**d**) Kinetics of gene expression in different endothelial cell subsets along the pseudotime scale. (**e**) Ratios of *MKI67*-positive cancer cells in all samples. (**f**) Correlation patterns between *MKI67* positive cancer cell ratio and endothelial cell percentage. Points were colored by BM, LM, and primary lung cancer. (**g**) mIHC staining of indicated genes to show the spatial relationship between blood vessels with angiogenesis features in BM.
