# Supplementary material for: Immunological and pathological characteristics of brain parenchymal and leptomeningeal metastases from non-small cell lung cancer
Source: Cell Discov. 2025 Aug 29;11:72. doi: 10.1038/s41421-025-00828-7 (PMC12397330; doi:10.1038/s41421-025-00828-7)
Supplement: Supplementary file 17 — Supplementary Fig. S8: Comparison of normal and CNSm condition BBB cells, related to Fig. 6. [file 41421_2025_828_MOESM17_ESM.pdf]

Supplementary Fig. S8

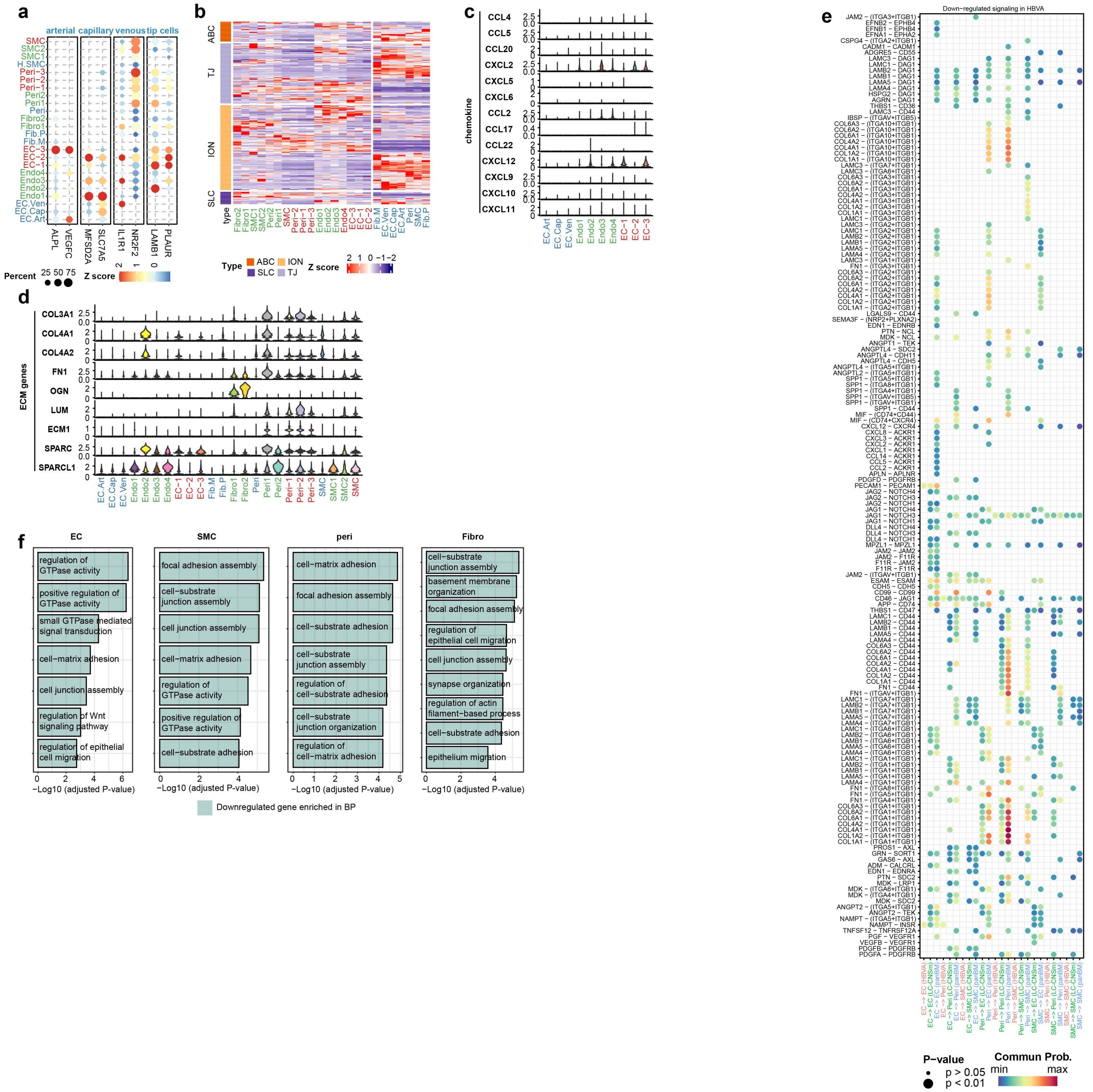

Supplementary Fig. S8: Comparison of normal and CNSm condition BBB cells, related to Fig. 6.

(**a**) Expression patterns of marker genes in 4 endothelial cell subtypes (arterial, capillary, venous, and tip cells) among BBB cells of 3 datasets. (**b**) Expression patterns of 4 functional pathways in BBB cells. Each row represented scaled gene expression, and each column represented one cell type. Abbreviations: ABC, TJ, ION and SLC mean ATP-binding cassette (ABC) transporters, tight junction, ion transporters, and solute carrier (SLC) transporters, respectively. (**c**) Expression patterns of chemokines in endothelial cells of 3 datasets, visualized by violin plot. (**d**) Expression patterns of representative extracellular matrix (ECM) genes in BBB cells of 3 datasets, visualized by violin plot. (**e**) Representative cell-cell interaction pairs of endothelial cells, smooth muscle cells, and pericytes in HBVA, panBM, and our LC-CNSm datasets. (**f**) Enriched GO BP terms using downregulated gene in Peri, SMC, EC, and Fibro in LC-CNSm-LM versus HBVA dataset.
