# Supplementary material for: Immunological and pathological characteristics of brain parenchymal and leptomeningeal metastases from non-small cell lung cancer
Source: Cell Discov. 2025 Aug 29;11:72. doi: 10.1038/s41421-025-00828-7 (PMC12397330; doi:10.1038/s41421-025-00828-7)
Supplement: Supplementary file 18 — Supplementary Fig. S9: Integration analysis among our data, published normal human brain parenchyma and meninges, related to Fig. 6. [file 41421_2025_828_MOESM18_ESM.pdf]

Supplementary Fig. S9

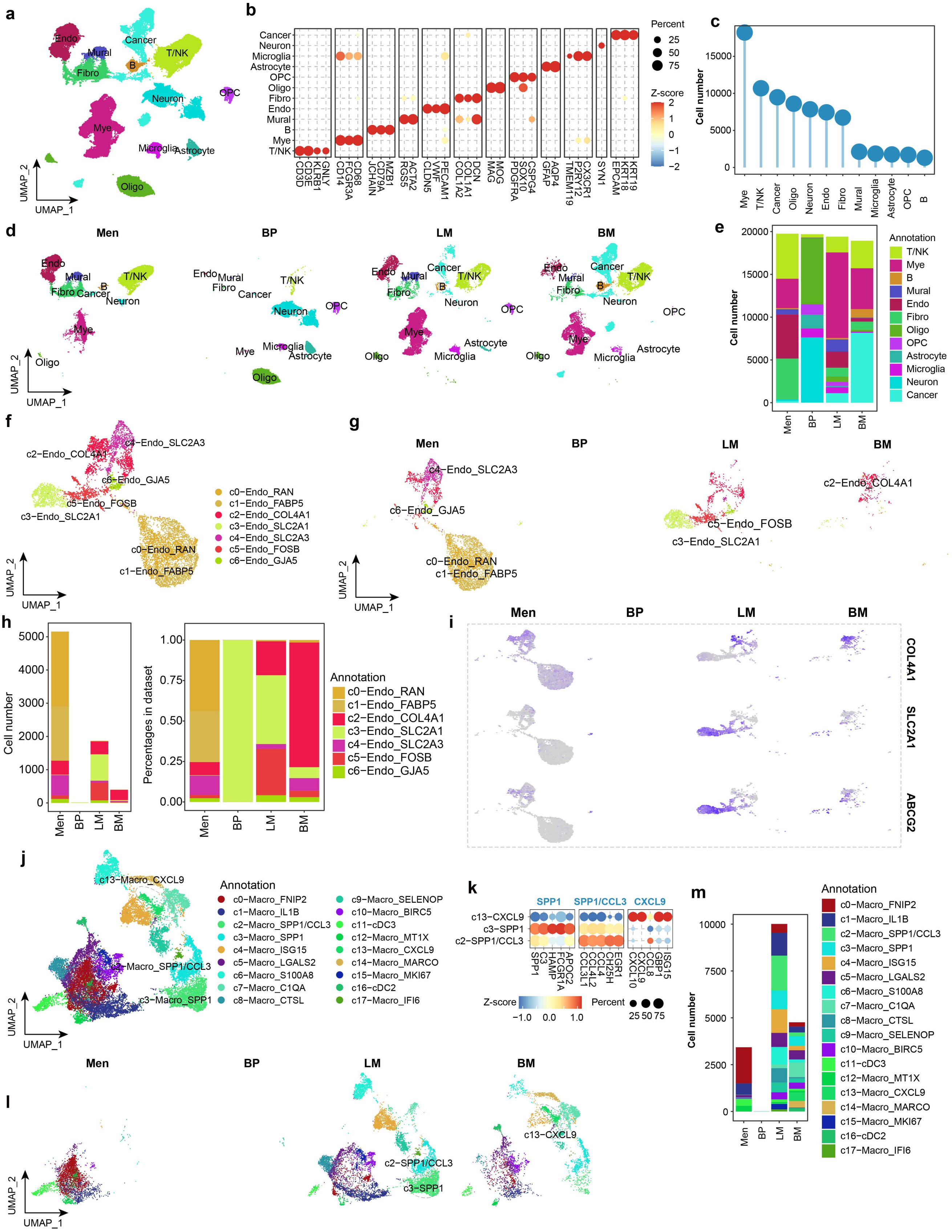

Supplementary Fig. S9: Integration analysis among our data, published normal human brain parenchyma and meninges, related to Fig. 6.

(**a**) UMAP visualization of main cell types from normal human brain parenchyma (BP) and meninges (Men), and our data. (**b**) The expression of marker genes for main cell types. (**c**) Cell numbers of each cell types. (**d**) UMAP visualization of main cell types, split by data source. (**e**) Main cell compositions in 4 data set, shown in cell number. (**f & g**) UMAP visualization (**f**) and data source (**g**) of endothelial cells, colored by endothelial cell clusters. (**h**) Endothelial cell compositions in 4 data set, shown in cell number (left panel) and percentages (right panel). (**i**) Feature genes (COL4A1 for Endo2, SLC2A1, ABCG2 for Endo1) expression pattern in endothelial cells. (**j**) UMAP visualization of myeloid cells. (**k**) Expression pattern of signature genes from Macro_CXCL9, Macro_SPP1, Macro_ SPP1/CCL3 cells in newly annotated myeloid clusters. (**l**) UMAP visualization of myeloid cells, split by data source. (**m**) Myeloid cell compositions in 4 data set, shown in cell number.
