# Supplementary material for: Immunological and pathological characteristics of brain parenchymal and leptomeningeal metastases from non-small cell lung cancer
Source: Cell Discov. 2025 Aug 29;11:72. doi: 10.1038/s41421-025-00828-7 (PMC12397330; doi:10.1038/s41421-025-00828-7)
Supplement: Supplementary file 19 — Supplementary Fig. S10: Graphical abstract of the study. [file 41421_2025_828_MOESM19_ESM.pdf]

Supplementary Fig. S10

a

Graphical abstract

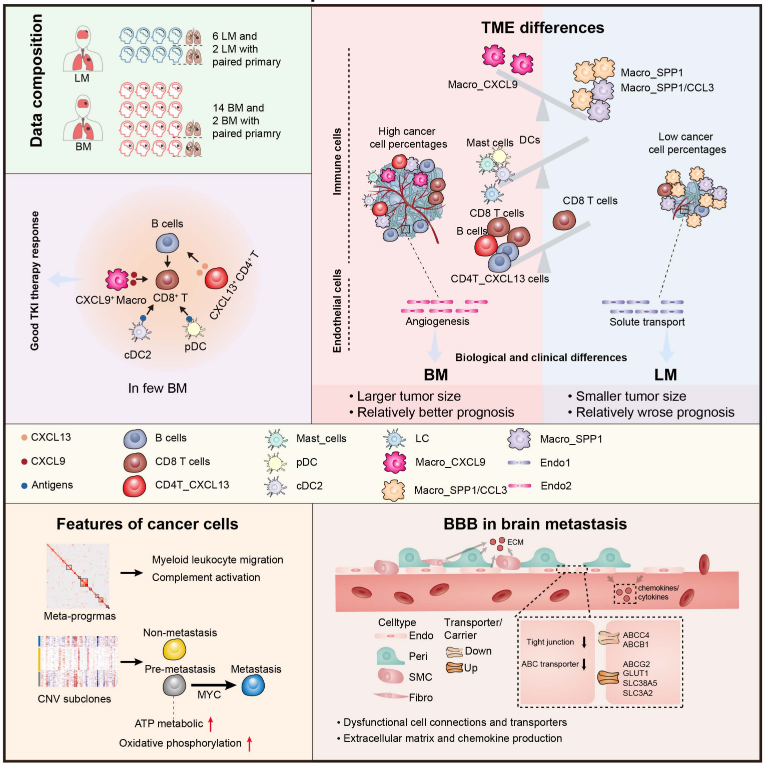

Supplementary Fig. S10: Graphical abstract of the study.

(**a**) Graphical summary of the study, including the dataset composition, main cell differences between BM and LM, and transcriptional changes associated with metastasis.
